# Inference of patient‐specific subpathway activities reveals a functional signature associated with the prognosis of patients with breast cancer

**DOI:** 10.1111/jcmm.13720

**Published:** 2018-07-04

**Authors:** Junwei Han, Siyao Liu, Ying Jiang, Chaohan Xu, Baotong Zheng, Minghao Jiang, Haixiu Yang, Fei Su, Chunquan Li, Yunpeng Zhang

**Affiliations:** ^1^ College of Bioinformatics Science and Technology Harbin Medical University Harbin China; ^2^ College of Basic Medical Science Heilongjiang University of Chinese Medicine Harbin China; ^3^ School of Medical Informatics Daqing Campus Harbin Medical University Harbin China

**Keywords:** pathway structure, patient‐specific, prognostic signature, subpathway activity

## Abstract

Breast cancer is one of the most deadly forms of cancer in women worldwide. Better prediction of breast cancer prognosis is essential for more personalized treatment. In this study, we aimed to infer patient‐specific subpathway activities to reveal a functional signature associated with the prognosis of patients with breast cancer. We integrated pathway structure with gene expression data to construct patient‐specific subpathway activity profiles using a greedy search algorithm. A four‐subpathway prognostic signature was developed in the training set using a random forest supervised classification algorithm and a prognostic score model with the activity profiles. According to the signature, patients were classified into high‐risk and low‐risk groups with significantly different overall survival in the training set (median survival of 65 vs 106 months, *P *=* *1.82e‐13) and test set (median survival of 75 vs 101 months, *P *=* *4.17e‐5). Our signature was then applied to five independent breast cancer data sets and showed similar prognostic values, confirming the accuracy and robustness of the subpathway signature. Stratified analysis suggested that the four‐subpathway signature had prognostic value within subtypes of breast cancer. Our results suggest that the four‐subpathway signature may be a useful biomarker for breast cancer prognosis.

## 1 INTRODUCTION

Breast cancer is one of the most deadly forms of cancer in women worldwide. It is increasingly being realized that breast cancer is extremely heterogeneous.^1^, ^2^ The identification of better prognosis biomarkers of the cancer is necessary for earlier diagnosis and more personalized treatment. A number of clinical prognostic factors, such as tumour node metastasis (TNM) stage, pathological grade and histologic type of the tumour, have been used to predict the outcome of cancer, but their predictive power is limited.^3^ Because of the development of high‐throughput experimental techniques, such as microarrays and next‐generation sequencing, many studies have investigated prognostic signatures at the molecular level.^4^ Some molecular signatures, such as the 70‐gene signature discovered by Netherlands Cancer Institute (NKI70),^5^, ^6^ performed well as predictors of survival in breast cancer. However, current studies have mainly prioritized biomarkers by detecting the correlations of gene expression with survival data and have poorly considered biological interactions and functions among genes.

Biological pathways are models containing structure information, such as interactions, regulation, modifications and binding, between genes. In addition, genes involved in the same pathway often perform a specific biological function together.^7^ Pathway databases, such as the Kyoto Encyclopaedia of Genes and Genomes (KEGG),^8^ provide useful pathway structure information. Many pathway identification methods, such as signalling pathway impact analysis (SPIA), have effectively uncovered dysregulated pathways underlying complex traits and human diseases.^9^ However, entire pathways are often too large to enable accurate interpretation of relevant biological phenomena. In recent years, key subpathway regions representative of the entire corresponding pathway are believed to be more useful in terms of interpreting relevant biological phenomena, and abnormalities of these subpathway regions may contribute to the aetiology of diseases.^10^, ^11^, ^12^, ^13^, ^14^, ^15^ Subpathway‐GM^10^ was proposed to identify disease‐relevant subpathways by integrating information from genes and metabolites and pathway structure information within the given pathway. In the study, 16 statistically significant subpathways were identified as associated with metastatic prostate cancer, of which a subpathway region of histidine metabolism, which was ignored by the entire pathway analysis method, was demonstrated to be associated with prostate cancer cell migration in dose‐dependent and time‐dependent manners. SubpathwayMiner^15^ used a subgraph mining method to find the subpathways in which all genes have highly similar functions, and 36 dysregulated subpathways enriched by differential expression genes were identified as associated with the initiation or progression of lung cancer. These studies showed that abnormal subpathways may play important roles in the progress of cancer.

Moreover, there are some other methods to identify subpathways from pathway topology. For instance, PATHOME (pathway and transcriptome information) first decomposed the pathways into linear paths (subpathways) from the top nodes to leaf nodes and then evaluated the significance of differential expression patterns between cancer and normal tissue along the subpathways. TEAK (Topology Enrichment Analysis frameworK) extracted linear and nonlinear subpathways with pathway topologies and scored them using the Bayes Net Toolbox to fit a context‐specific Gaussian Bayesian network for each subpathway. MinePath facilitated the decomposition of pathways into their constituent subpathways, and then, the subpathways were matched with gene expression sample profiles in order to evaluate their functional status and to assess phenotype differential power. These subpathway analysis methods have mainly identified dysregulated subpathways by comparing the expression levels of their involved genes between tumour and normal tissues and then analysing the performance of the methods. In this way, patient‐specific clinical or prognosis status of the subpathway is lost before the outcomes are obtained. Identification of prognosis‐related subpathways may help to predict the survival of patients with cancer and may provide information for personalized therapy.

This study aimed to infer patient‐specific subpathway activities for revealing a functional signature associated with the prognosis of patients with breast cancer. We identified a four‐subpathway signature with the ability to predict the overall survival of patients with breast cancer and validated its prognostic value in five independent breast cancer data sets.

## 2 MATERIALS AND METHODS

### 2.1 Data summary

We collected six independent breast cancer data sets of 1502 patients. We first used the gene expression data set of 255 breast cancer patients in van de Vijver et al.^6^ to identify the prognostic signature. Microarray expression profiling was performed using Agilent microarray technologies. The data set is frequently used in breast cancer studies as it contains abundant clinical characteristics, including age, tumour size, lymph node status, grade and oestrogen receptor status. The patients were randomly divided into a training set and (n = 147) and a test set (n = 148) with almost the same number. We then enrolled five other independent breast cancer data sets (GSE7390,^16^ GSE1456,^17^ GSE3143,^18^ GSE1992^19^ and gene expression array in TCGA^20^) to validate the prognostic signature. A total of 1207 patients were enrolled in these data sets, and each data set included more than 150 patients. Gene expression profiles were measured by different microarray platforms. We downloaded the clinical characteristics of patients in each set (if provided). For all of the above data sets, the summary of patients and clinical characteristics was listed in Table S1. The breast cancer patients in the training sets were assigned into groups with poor or good prognosis according to the status of patient death or not. In this study, the gene expression levels of the data sets were log2 transformed which may cause the raw data set to be approximate normal distribution. And then the gene expression data were *z*‐score normalized across arrays.

For pathway data, we collected 236 biological pathways from the KEGG database,^8^ which contained experimentally verified pathway structure information (eg, interactions, regulation, modifications and binding between genes). For each pathway, we converted the data into an undirected and unweighted gene‐gene network on the basis of pathway structure information using the “iSubpathwayMiner” system.^10^, ^15^

### 2.2 Inference of patient‐specific subpathway activities

**Figure 1:**
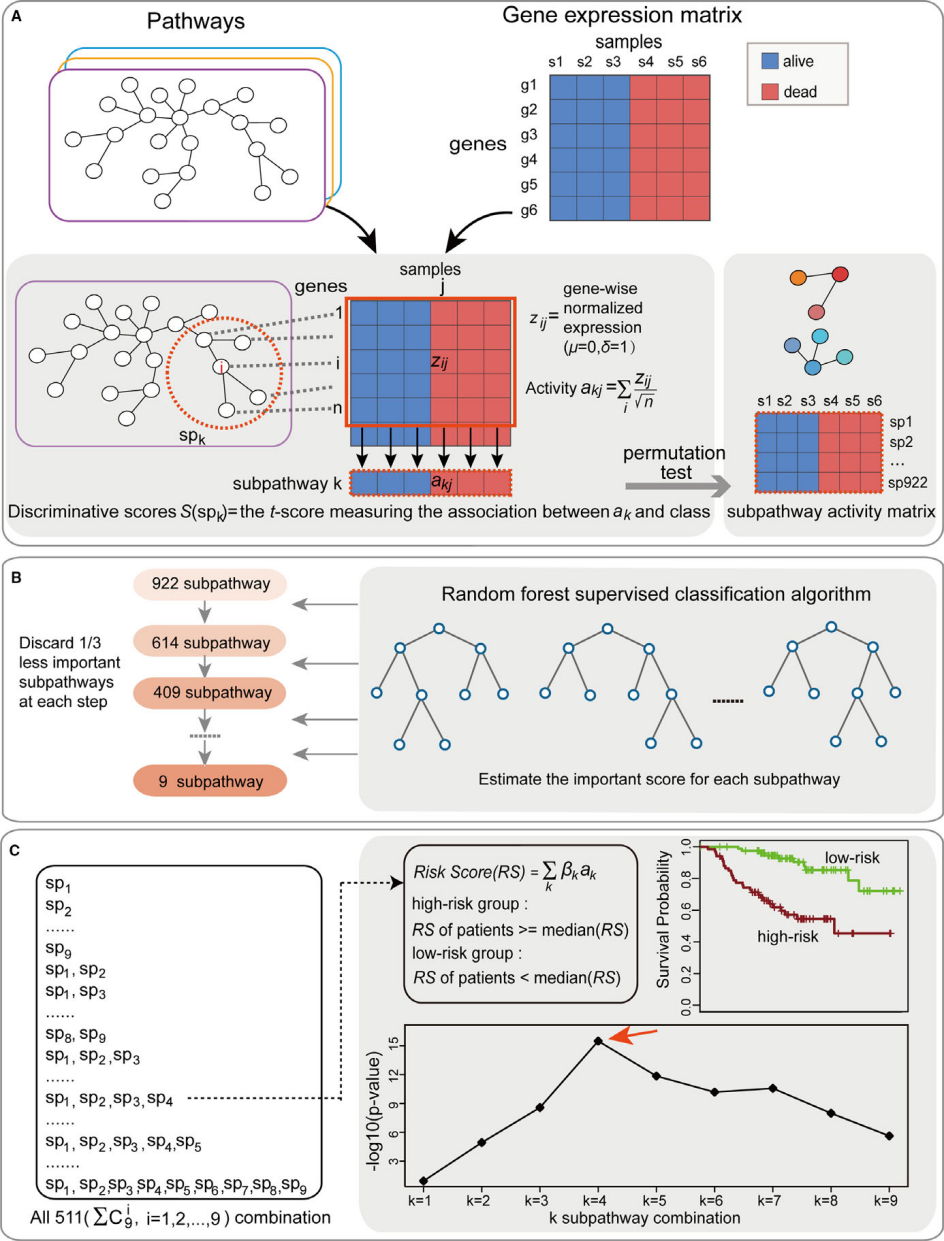
Identification of the subpathway signature in the training set. A, Gene expression profiles of tissue samples with phenotypes of good or poor prognosis were transformed into a “subpathway activity matrix.” For a given subpathway *sp*_*k*_ in a certain pathway network, the activity was a combined z‐score derived from the expression of its individual genes. After overlaying the expression vector of each gene on its corresponding protein in the pathway network, subpathways with discriminative activities were identified via a greedy search algorithm. Based on permutation tests, the subpathway activity matrix of significant subpathways was obtained. B, The random forest supervised classification algorithm was used to identify the subpathways that were most related to prognostic classifications. An iteration procedure was implemented to narrow down the subpathway sets by discarding one‐third of the least important lncRNAs at each step according to their importance score, and nine subpathways remained. C, Development of a prognostic classifier for all combinations of the nine subpathways using the risk score model. For each subpathway combination, patients were classified into high‐risk and low‐risk groups according to median risk scores, and the log‐rank test was used to evaluate the performance of classifications. The signature with the largest value of −log(*p*) was selected as the final signature

A subpathway was defined as a local region that induces a single connected component in the entire pathway. The subpathways are believed to be more useful in terms of interpreting the relevant biological phenomena, and abnormalities of subpathway regions may contribute to the progression of diseases.^10^ We present a novel method that integrates pathway structure information with gene expression data to infer patient‐specific subpathway activities. The expression values of each gene were overlaid on its corresponding gene in the pathways to integrate the pathway structure with gene expression. We searched for the subpathways within each pathway whose combined expression levels across the samples were highly discriminative of the status of cancer patients (Figure 1A). For a particular subpathway *sp*_*k*_, we used *a*_*k*_ to denote its vector of activity scores for the cancer patients in a study and used *c* to denote the corresponding vector of class labels (eg, good vs poor prognosis). The activity vector *a*_*k*_ was derived by normalizing the gene expression values *g*_ij_ to z‐transformed scores *z*_ij_; for each gene, *i* has a mean μ_*i*_ = 0 and a standard deviation σ_*i*_ = 1 over all samples *j*. The *j*th element of *a*_*k*_ corresponds to the subpathway activity of sample *j*, and the individual *z*_ij_ of each member gene in the subpathway is averaged into a combined *z*‐score with formula (1):

(1) $$a_{\mathrm{kj}}=\sum_{i}\frac{Z_{\mathrm{ij}}}{\sqrt{n}}$$

where *n* is the number of genes involved in the subpathway. The discriminative potential of a candidate subpathway was evaluated by many types of statistics; we defined the discriminative score *S*(*P*_*k*_) as the *t*‐test statistic derived on the activity vector *a* between the groups of samples distinguished by the class label vector *c*.

For each given pathway, a greedy search algorithm as previously described^21^ was performed to identify subpathways within the pathway network for which the discriminative scores *S*(sp_*k*_) were locally maximal. The search algorithm starts from a seeded gene *i* and expands iteratively. At each iteration, the search algorithm considers addition of a gene from the neighbours of genes in the current subpathway. The additional gene is adopted if it yields a maximal score increase. The search will terminate and output candidate subpathways when no additional gene increases the score over a specified improvement rate *r* in which (1 + *r*) × *S*(*P*_*k*_) to avoid over‐fitting the expression data. In this study, we set *r* at 0.05. Furthermore, only subpathways with more than three genes and less than 50 genes were retained, which avoids overly narrow or broad functional subpathways.

Three tests of significance are performed to evaluate the statistical significance of the subpathways identified in the search step. For the first test, we performed a gene‐based permutation test that permutes gene labels across all the pathway networks and preserves the gene expression profiles and prognosis status in the data set. We then recomputed the discriminative scores for each real subpathway. This permutation was used to test the correlation between real subpathways and pathway structure. We performed 10 000 permutations for this test. Second, we recomputed the scores for each real subpathway over 1000 random trials in which the prognosis status vector was randomly permuted. This permutation was used to test the associations between real subpathways and prognosis status. The third test was used to test whether the real subpathways were statistically significant within their corresponding entire pathways. For each real subpathway, we randomly extracted the same number of genes, which initialized from the same seed gene as the real subpathway, from the entire pathway and recomputed the subpathway score. The real subpathway score was compared with 1000 random scores. For each test (*i *=* *1,2,3), the statistically significant level of a subpathway was calculated as *P *= *M*/*N*, where *M* is the number of permuted subpathway scores greater than the real subpathway score and *N* is the permutation times. We adjusted *P* using the false discovery rate (FDR) method proposed by Benjamini and Hochberg^22^ to correct for multiple comparisons. Because of different number of times for the three permutation tests, we chose different FDR cut‐offs. The significant subpathways are selected that satisfy all three tests with FDR_1_ < 0.0001, FDR_2_ < 0.001 and FDR_3_ < 0.001. In training set, we obtained 922 significant subpathways. The subpathway activity matrix was used for further analysis.

### 2.3 Selection of subpathways mostly related to prognostic classification

Based on the activity matrix of 922 significant subpathways, a random forest supervised classification algorithm was used to identify subpathways mostly related to prognostic classifications (Figure 1B). The reason for using the random forest algorithm is that it could incorporate interactions between subpathways and return measures of subpathway importance. In the algorithm, we implemented an iteration procedure to narrow down the subpathway set in which the least important subpathways were discarded at each iteration step. This strategy has been successfully used for subtype predictions of breast cancer based on gene expression profiles.^23^ In detail, we constructed ten thousand trees at each iteration step and set the square root of the number of input subpathways to the size of randomly chosen subpathways at each node of the single classification tree. The important score for each subpathway was estimated on out‐of‐bag samples by permutation testing, and one‐third least important subpathways were discarded at each step. After each discard, we re‐estimated the generalization error of the classification on out‐of‐bag samples. We found that the generalization error changed slightly at first, but it increased sharply when less than nine subpathways were retained (Figure S1). Thus, nine subpathways mostly related to prognostic classifications were selected among the 922 significant subpathways. Detailed information on the nine subpathways is listed in Table S2. Previously, this algorithm was successfully applied to identify the lncRNAs that are most related to oesophageal cancer.^24^ We implemented the algorithm with the R package ranger.^25^

### 2.4 Identification of the prognostic subpathway signature

We developed a signature by selecting a combination of the nine subpathways from the training set (Figure 1C). There are 511 ($\sum C_{9}^{i}$, *i *=* *1, 2, …,9) combinations of the nine subpathways. For each combination, we calculated the risk score for every patient from the activities of the subpathways by using a prognostic score model 26, 27 as follows:

$$\text{Risk}\;\;\text{score}=\sum_{k\in S}\beta_{k}a_{k}\;\left(2\right)$$

where *S* is one of 511 combinations; *a*_*k*_ is the activity of subpathway *k;* β_*k*_ is the regression coefficient of a univariate Cox proportional hazard regression model estimated on *a*_*k*_ and the overall survival data. A high‐risk score indicates poor survival for patients. According to the median of risk scores, patients in training set were classified into high‐risk and low‐risk groups for each combination. We used the Kaplan‐Meier method to evaluate the power of classification for each combination to determine which combination is optimum for prognostic classification in the training set. For signatures consisting of a specific number of subpathways (*i *=* *1, 2, …, 9), the subpathway with the smallest *P* in the log‐rank test was selected for each *i* (Figure 1C). After the results were compared, a four‐subpathway signature was then defined as the final signature.

## 3 RESULTS

### 3.1 Derivation of a four‐subpathway prognostic signature

For prognostic signature analysis, the147 breast cancer patients in the training set were first assigned into groups with poor (41 patients) or good (106 patients) prognosis according to the status of patient death or not. We integrated the pathway structure with gene expression to infer the patient‐specific subpathway activities that were associated with overall patient survival (Figure 1A). In total, 4531 candidate subpathways were identified using a greedy search algorithm. Through the three different permutation analyses, 922 significant subpathways were obtained with the default threshold (Materials and methods). A heat map of the activities of 922 subpathways displayed clear differences between patients with good prognosis and poor prognosis (Figure S2). The random forest supervised classification algorithm was applied to identify the subpathways most related to prognostic classifications (Figure 1B). Nine subpathways were selected among the 922 significant subpathways.

We developed subpathway signatures that were constructed by any combination of the nine subpathways (Figure 1C). In total, there were 511 candidate signatures (Materials and methods). For each candidate signature, we calculated the risk score for every patient based on the activities of the subpathways using a prognostic score model. In the training set, patients were classified into high‐risk and low‐risk groups according to median risk scores. We further prioritized signatures using the Kaplan‐Meier method and log‐rank test to derive an optimum subpathway signature to predict overall patient survival. A four‐subpathway signature with the best classification results (the smallest *P* in log‐rank test) was selected to construct the final signature. Through univariate Cox proportion hazard regression analysis, the activities of three subpathways (path:04390_17 in hippo signalling pathway, path:04730_1 in long‐term depression, and path:00230_30 in purine metabolism) in the four‐subpathway signature were positively associated with overall survival, and the activity of the fourth subpathway (path:04151_102 in PI3K‐Akt signalling pathway) was inversely associated with overall survival. Detailed information on each subpathway is listed in Table 1.

**Table 1:** Detail information for the four subpathways in the signature

SubpathwayID	Pathway	Size	HR (95% CI)	*P*‐value	Genes
path04390_17	Hippo signalling pathway	8	2.75 (2.0‐3.79)	4.83E‐10	YWHAZ, YWHAG, YAP1, SOX2, SERPINE1, TEAD3, BIRC5, FGF1
path04730_1	Long‐term depression	10	2.12 (1.65‐2.70)	2.59E‐09	LYN, PRKCA, PLA2G4B, GNAS, GNAZ, GNA12, CRHR1, GRM1, GNAQ, GNAI1
path04151_102	PI3K‐Akt signalling pathway	9	0.40 (0.27‐0.56)	1.20E‐07	GH2, JAK2, IL2RG, PIK3CD, IRS1, IL7R, IGF1R, FGF10, FGF18
path00230_30	Purine metabolism	8	2.38 (1.76‐2.30)	1.40E‐08	PDE1A, GMPS, ITPA, POLR3A, PDE2A, ENTPD2, RRM2, ADCY7

HR, hazard ratio; CI, confidence intervals.

We derived a formula to calculate the risk score of the signature for every patient from the activities of the four subpathways weighted by the univariate Cox proportional hazard regression coefficient^26^, ^27^:

Risk score=1.013×activity of path:04390_17+0.750×activity of path:04730_1+0.866×path:00230_30−0.927×activity of path:04151_102(3)

With this risk score formula, a patient in the training set was classified as high risk if the risk score was higher than the median risk score (0.1067) and as low risk if was not.

### 3.2 A four‐subpathway signature predicts overall survival of patients with breast cancer

**Figure 2:**
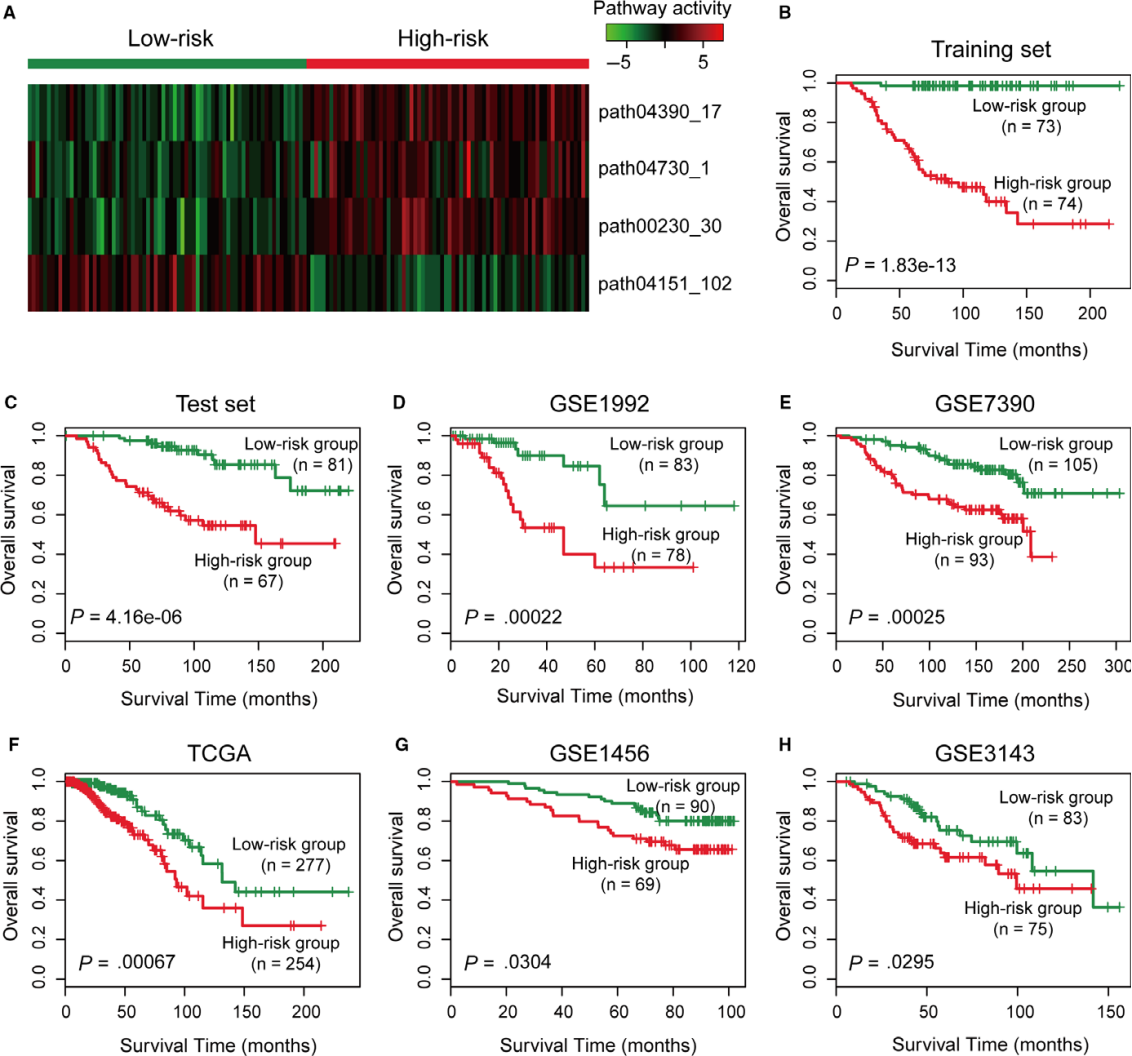
The four‐subpathway signature predicts overall survival of patients with breast cancer. A, Heatmap of the activities of four subpathways of the signature in the training set. B‐H, Kaplan‐Meier survival curves of patients classified into high‐ and low‐risk groups using the four‐subpathway signature in the training, test, GSE1992, GSE7390, TCGA, GSE1456 and GSE3143 data sets, respectively. *P* was calculated by log‐rank test. Vertical hash marks indicate censored data

Patients in the training set were divided into a high‐risk group (n = 74) or a low‐risk group (n = 73) using the four‐subpathway signature with the median risk score as the cut‐off. The activities of four subpathways in the signature display obviously differences between patients in the high‐risk group and those in the low‐risk group (Figure 2A). Patients with high‐risk scores had shorter overall survival than patients with low‐risk scores (median survival of 65 vs 106 months, *P *=* *1.82e‐13 in log‐rank test; HR = 1.90, 95% CI 1.57‐2.30, *P* = 5.10e‐11in univariate Cox proportion hazard regression analysis) (Figure 2B and Table S3). Five clinical characteristics (age, tumour size, LN status, tumour grade and ER status) were provided in the training set. The distributions of age, tumour grade and ER status differed significantly, but the distributions of other clinical factors did not (Table 2).

**Table 2:** Clinical and pathological characteristics of patients with breast cancer with high‐ or low‐risk subpathway signature in the training and test sets

Characteristics	Training set (N = 147)			Test set (N = 148)		
	High‐risk group (n = 74)	Low‐risk group (n = 73)	*P*‐value	High‐risk group (n = 67)	Low‐risk group (n = 81)	*P*‐value
Age, median (sd)	44.5 (6.32)	46 (4.56)	.014^a^	43 (5.64)	45 (5.08)	.38^a^
Tumour size (%)						
≤2 cm	32 (43.24)	42 (57.53)	.12	31 (46.27)	50 (61.73)	.086
>2 cm	42 (56.76)	31 (42.47)		36 (53.73)	31 (38.27)	
LN status (%)						
Positive	40 (54.05)	39 (53.42)	1	32 (47.76)	33 (40.74)	.49
Negative	34 (45.95)	34 (46.58)		35 (52.24)	48 (59.26)	
Grade, No. (%)						
1	7 (9.46)	30 (41.1)	<.001	5 (7.46)	33 (40.74)	<.001
2	18 (24.32)	26 (35.62)		22 (32.84)	35 (43.21)	
3	49 (66.22)	17 (23.28)		40 (59.70)	13 (16.05)	
ER status (%)						
Positive	43 (58.11)	67 (91.78)	<.001	37 (55.22)	79 (97.53)	<.001
Negative	31 (41.89)	6 (8.22)		30 (44.78)	2 (2.47)	
Median survival (months)	65.15	106.33	<.001^b^	75.07	101.8	<.001^b^

LN, lymph node; ER, oestrogen receptor.

*P*‐values are calculated by chi‐square test, unless otherwise stated.

a Student's *t* test.

b Log‐rank test.

We applied the same formula and cut‐off point as those derived from the training set to a test set of 148 patients to test the prognostic value of the four‐subpathway signature. The signature classified 67 and 81 patients into the high‐risk and low‐risk groups, respectively. As expected, the overall survival time of high‐risk group patients was significantly shorter than that of low‐risk group patients (median survival of 75 vs 101 months, *P *=* *4.17e‐5 in log‐rank test; HR = 1.25, 95% CI 1.13‐1.38, *P* = 9.12e‐06 in univariate Cox proportion hazard regression analysis) (Figure 2C and Table S3). The clinical characteristics of tumour grade and ER status were significantly different, whereas the other clinical characteristics (age, tumour size and LN status) were not significantly different between high‐ and low‐risk group patients (Table 2).

We used the prognostic value of the signature to classify patients from the five independent breast cancer data sets (GSE1992, GSE7390, GSE1456, GSE3143 and gene expression array in TCGA) to validate whether the four‐subpathway signature had the same or similar prognostic value in different populations (Table S1). For each independent set, patients were divided into high‐risk and low‐risk groups, and patients with high‐risk scores had significantly shorter overall survival than those with low‐risk scores (Figure 2D‐H and Table S3). Specifically, the *P* of log‐rank tests in the data sets of GSE1992, GSE7390 and TCGA were all less than 0.001 (Figure 2D‐F). For the data sets of GSE1456 and GSE3143, the *P* of log‐rank tests were less than 0.05 (Figure 2G and H). For univariate Cox proportion hazard regression analysis, the HRs of all five validated sets were more than 1.2, and *P* was less than 0.01 (Table S3). These results indicate that the subpathway signature can be used for classifying patients with breast cancer. Moreover, three data sets (GSE1992, GSE7390 and TCGA) provided various clinical characteristics, and we analysed the differences in clinical characteristics between high‐ and low‐risk group patients (Table S4). ER status differed significantly between the two groups across three data sets, and tumour grade differed significantly between the two groups in the GSE1992 and GSE7390 data sets.

### 3.3 Survival prediction by the four‐subpathway signature is independent of other clinical characteristics

We then assessed whether the survival prediction ability of the four‐subpathway signature was independent of the other clinical characteristics of patients with breast cancer. Multivariate Cox regression analysis was performed using a backward stepwise variable selection approach. Five data sets (training set, test set, GSE1992, GSE7390 and TCGA) that included clinical characteristics were adopted. The clinical characteristics provided by the different data sets varied; therefore, the four‐subpathway signature and other clinical characteristics (such as age, tumour size, tumour grade, LN status and ER status) of each data set were used as covariates. Because some clinical characteristics (eg, LN status, ER status and HER2 status) were missing for some patients in the GSE1992 and TCGA data sets, we used the model of classification and regression trees^28^ implemented by the R package rpart to impute the missing values of clinical characteristics in the multivariate Cox regression analysis. The results showed that the four‐subpathway signature was an independent prognostic factor for overall survival across four data sets, except for the test set (Table S5). Meanwhile, in the test set, the *P* of the subpathway signature (*P *=* *.0503) has just exceed the significance threshold of 0.05.

### 3.4 The four‐subpathway signature has prognostic value within subtypes of breast cancer

**Figure 3:**
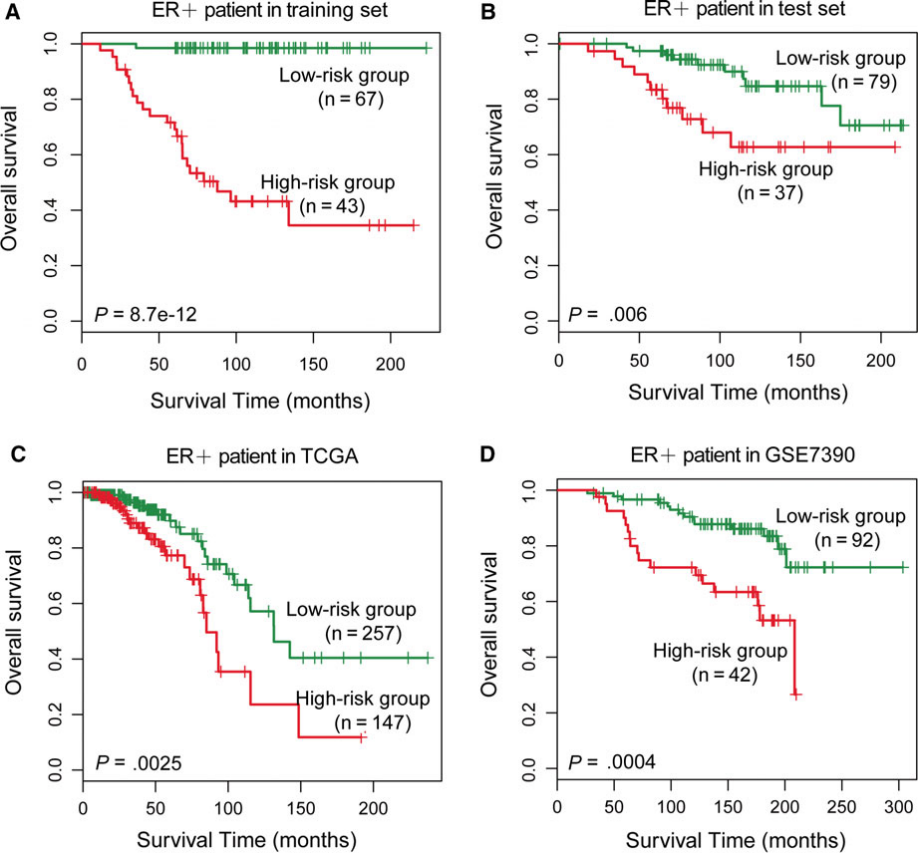
Survival prediction in ER+ patients. Kaplan‐Meier survival curves of ER+ patients with breast cancer classified into high‐ and low‐risk groups based on the four‐subpathway signature. A, Training set (n = 110). B, Test set (n = 116). C, TCGA (n = 404). D, GSE7390 (n = 64). Vertical hash marks indicate censored data

ER status of breast cancer is important clinically and is used both as a prognostic indicator and as a treatment predictor.^29^ Breast cancers can be classified as ER‐positive (ER+) and ER‐negative (ER−) subtypes based on their ER status. We next carried out a stratified analysis in ER+ and ER− patients to evaluate whether the four‐subpathway signature could predict the survival of patients within the same subtype. Log‐rank tests of ER+ patients in both the training set (*P *=* *8.7e‐12, Figure 3A) and the test set (*P *=* *.006, Figure 3B) showed that the subpathway signature could classify ER+ patients with cancer into high‐ and low‐risk groups. Additionally, the four‐subpathway signature showed similar prognostic value for the ER+ patients in the TCGA (*P *=* *.0025, Figure 3C) and GSE7390 (*P *=* *.0004, Figure 3D) data sets. In clinical practice, patients with ER+ breast cancer have a relatively good prognosis for 5‐year survival,^30^ but there are still some high‐risk patients in this cohort. The subpathway signature may help doctors identify high‐risk patients in the ER+ cohort and perform adjuvant chemotherapy.

For patients with ER− breast cancer, the four‐subpathway signature classified the patients into high‐ and low‐risk groups in the training set (*P* = .029, Figure S3A) but not in the test set (*P* = .15, Figure S3B). As with the test set, the four‐subpathway signature showed similar prognostic value for ER− patients in the TCGA (*P* = .49, Figure S3C) and GSE7390 (*P* = .8, Figure S3D) data sets. Patients with ER− generally have a relatively poor prognosis.^31^ The subpathway signature classified 83.8%, 93.8%, 87.3% and 79.7% of patients with ER− into high‐risk groups in the four data sets. In comparison, 45.9%, 53.1%, 18.6% and 39% of patients with ER− have poor prognosis in the data sets. This may result in a non‐significant *P* in the log‐rank test.

We also did stratified analyses of patients with HER2+ and HER2− patients from the TCGA and GSE1992 data sets, and obtained similar results (see Appendix S1 and Figure S4).

### 3.5 Comparison of survival prediction power between clinical characteristics and the four‐subpathway signature

**Figure 4:**
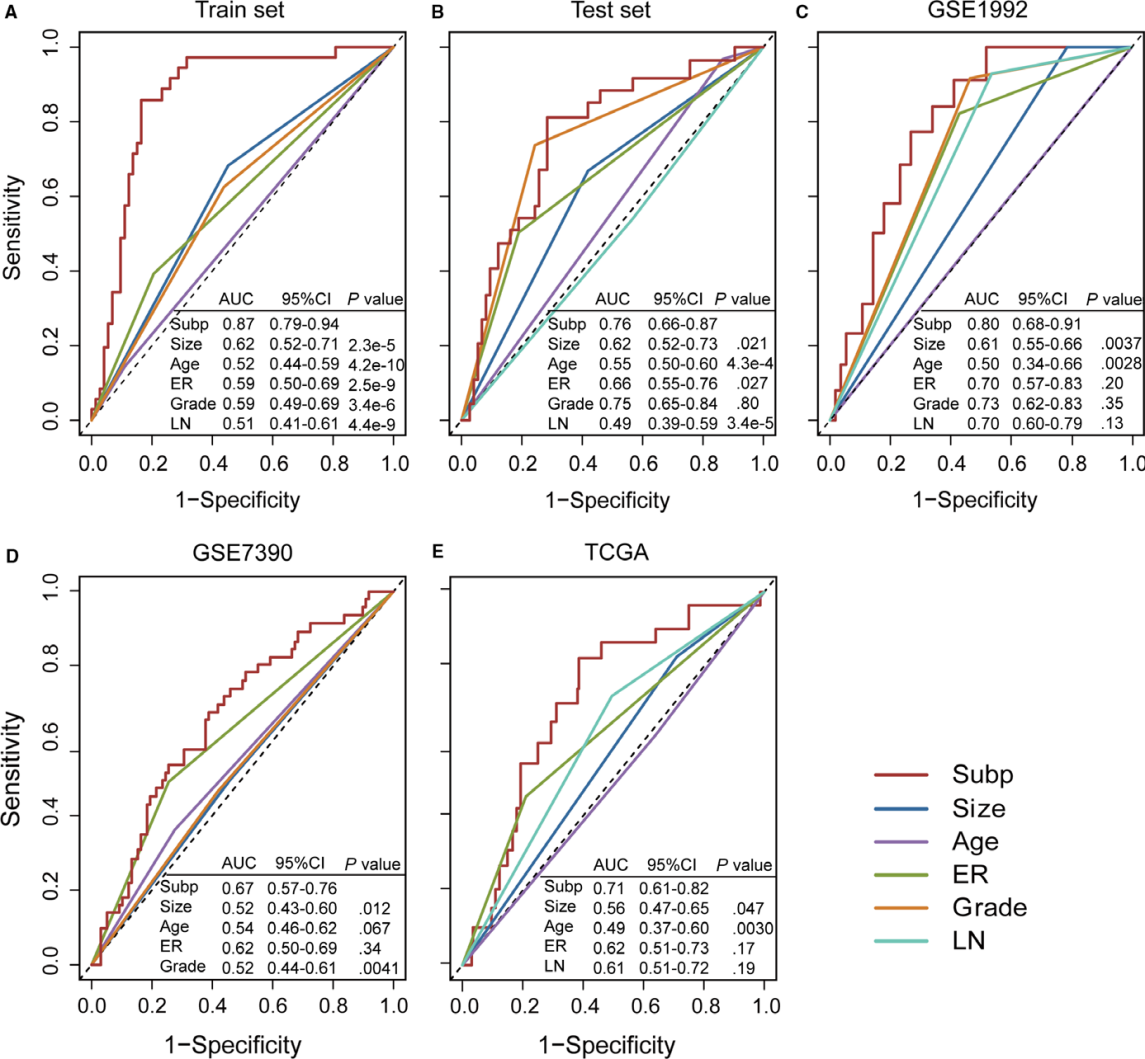
Comparisons of the sensitivity and specificity for the prediction of overall survival by the four‐subpathway signature (Subp), tumour size (Size), age, ER status, grade and LN status in patients with breast cancer. Receiver operating characteristics (ROC) curves in the (A) training set, (B) test set, (C) GSE1992, (D) GSE7390 and (E) TCGA. *P*‐values show the area under the ROC (AUC) of four‐subpathway signature versus the AUC of tumour size, age, ER status, grade and LN status

We performed time‐dependent ROC analysis^32^ to compare the sensitivity and specificity in survival prediction between the four‐subpathway signature and clinical characteristics (age, tumour size, tumour grade, LN status and ER status) in the five data sets that provided clinical characteristics (training set, test set, GSE1992, GSE7390 and TCGA). For each data set, median follow‐up time was used in time‐dependent ROC analysis. The *P* of the comparison test of the area under the ROC (AUC) of the four‐subpathway signature versus the AUC of tumour size, age, ER status, tumour grade and LN status were calculated using the R package time ROC.^32^ The four‐subpathway signature showed better prediction of survival than age and tumour size in regard to overall survival across the five data sets (*P* < .05, Figure 4A‐E). The predictive ability of the four‐subpathway signature was significantly different in the training set and test set (*P* = 2.54e‐09 and *P* = .027, Figure 4A and B) compared with the predictive ability of ER status, but it did not show a significant difference in the GSE1992, GSE7390 and TCGA data sets (*P* > .05; Figure 4C‐E). Meanwhile, there was no significant difference in these measures between the four‐subpathway signature and tumour grade in the test set and GSE1992 set (Figure 4B and C).

### 3.6 All four subpathways of the signature are essential for its prognostic value

We constructed all possible signatures containing one to four subpathways in our signature to confirm that all four subpathways of the signature were essential for the four‐subpathway signature. After the survival of these newly constructed signatures was compared with the original four‐subpathway signature using the log‐rank test, none of the signatures with fewer than four subpathways was consistently associated with overall patient survival in all seven data sets in our study (see Table S6). Furthermore, we tested the correlation between each pair of the four subpathways in the signature. The results showed that the maximum Pearson correlation coefficient between the four subpathway activities is less than 0.6 (Figure S5). These results indicate that all four subpathways are essential for the prognostic value of the signature.

### 3.7 Comparison of the prognostic power of the four‐subpathway signature and a gene‐based signature

We then compared the prognostic power of the subpathway‐based signature and a gene‐based signature on overall survival. The NKI70 gene signature (MammaPrint)^5^ is one of the most commonly used gene‐based models for breast cancer prognosis prediction, and it has been approved by the Food and Drug Administration for use in clinics. We applied the NKI70 method^6^ to the seven data sets used in our study and compared the MammaPrint gene signature with our subpathway‐based signature. Specifically, we mapped the 70 genes in the MammaPrint signature to the expression profile of each data set and calculated the correlation coefficient of the expression levels of these 70 genes with their average profile in tumours from patients with a good prognosis in the NKI study. As performed in the NKI study, the patients in each data set were classified into high‐risk or low‐risk groups according to the correlation coefficient (<0.4 or >0.4). The Kaplan‐Meier method and log‐rank tests were used to compare the results of the four‐subpathway signature and those of the MammaPrint gene signature. In the training set and test set, the MammaPrint signature gave log‐rank test *P* of .005 and .032 for overall survival analysis, respectively; in contrast, the four‐subpathway signature gave *P* of 1.82e‐13 and 4.16e‐6 for the test and training sets, respectively (Figure S6). For the five independent validation sets, all the log‐rank test *P‐*values of the four‐subpathway signature were superior to those of the MammaPrint signature (Figure S6).

We also used time‐dependent ROC analysis to compare the prognostic power of the four‐subpathway signature with the MammaPrint gene signature. The results showed that the predictive performance of the four‐subpathway signature was significantly higher than that of the MammaPrint gene signature in the training set (AUC: 0.867 vs 0.766, *P* = .013), GSE1992 (AUC: 0.796 vs 0.613, *P* = .0038) and GSE7390 (AUC: 0.662 vs 0.557, *P* = .034) (Figure S7A,C,D). For the other four data sets (test set, GSE1456, GSE3143, TCGA), the predictive performance was quite consistent between the four‐subpathway signature and the MammaPrint gene signature (Figure S7B,E‐G). Moreover, there were a total of 35 genes in the four‐subpathway signature whose gene number was only half of that in the MammaPrint signature. Thus, our subpathway signature used fewer genes but yielded the same or better results than the MammaPrint signature.

### 3.8 Functional analysis of the four‐subpathway signature

**Figure 5:**
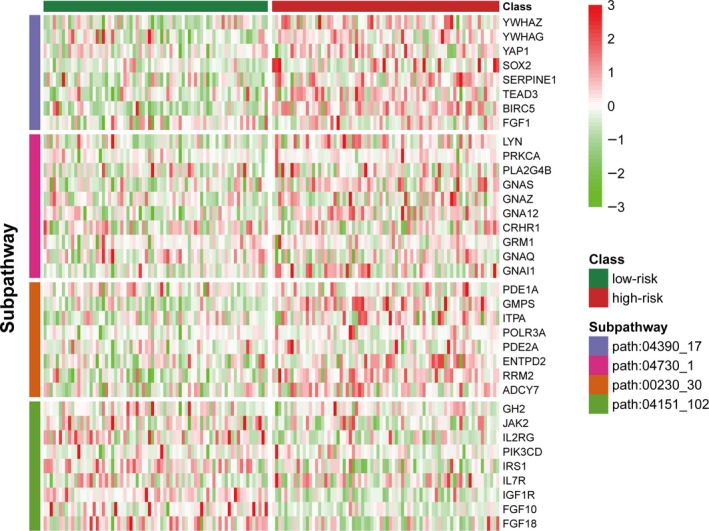
Heatmap of the expression levels after z‐score transformation for the genes involved in the four‐subpathway signature in the training set

The samples were divided into high‐risk and low‐risk groups according to the four‐subpathway signature. For the signature, the activities of the four subpathways displayed significant differences between the high‐risk and low‐risk groups in the training set (*t‐*test *P *<* *.05, Figure 2A). We then investigated the changes of genes involved in the four subpathways in the context of gene expression data. Also, the expression levels of the involved genes displayed significant differences between high‐risk and low‐risk groups (Figure 5). This result indicates that the subpathways in the signature may have an important biological function associated with the survival of patients with cancer.

We next sought to explore the biological function of the subpathways of the prognostic signature in breast cancer tumorigenesis and development. For path:04390_17 in the hippo signalling pathway, genes in the subpathways were mapped to the original pathway, and a local region was identified (Figure S8). This local region corresponds to key pathway downstream transcription co‐activators: YAP/TAZ, which has been reported to be associated with regulating cell growth, differentiation and apoptosis.^33^, ^34^ Specifically, elevated yes associated protein 1 (YAP1) activity has been correlated to a poor prognosis in several cancers,^35^, ^36^ TEA domain transcription factor 1 (TEAD1) has been shown to be the major YAP1 partner in breast cancer cell lines,^37^ and fibroblast growth factor 1 (FGF1) plays an important role in the regulation of cell survival, cell division, angiogenesis and cell differentiation.^38^ The other three subpathways have also been reported to be associated with the development of breast cancer (see Appendix S1 and Figures S9, S10, S11).

## 4 DISCUSSION

Breast cancer is increasingly recognized as being highly heterogeneous.^1^, ^2^ Routine clinical practice, such as the TNM staging system, is not adequate for breast cancer prognosis. However, prognostic assessment is crucial for earlier diagnosis and more personalized treatment. Recently, the identification of molecular signatures with high‐throughput biological data was a promising approach to predicting the prognosis of patients with cancer.^5^ However, a common problem with using high‐throughput data is the “curse‐of‐dimensionality” problem with many more genes than sample numbers. We overcame this problem by incorporating gene expression data into pathway structure and using a greedy algorithm to search the subpathways associated with the prognosis of patients. Although a number of methods for identifying subpathways associated with cancer have been proposed,^10^, ^11^, ^12^, ^13^, ^14^, ^15^ the advantage of our method is that it considered patient‐specific clinical and prognosis information in the identification of subpathways, and constructed subpathway activity profiles for each patient in the data set.

The prognostic value of the four‐subpathway signature was verified in the test set and five independent validation sets. The gene expression profiles of these data sets were measured by five different platforms of two chip company (Agilent and Affymetrix) (Table S1). The four‐subpathway signature was found to robustly predict the survival of patients with breast cancer from the above data sets. The good prognosis prediction of the signature in these data sets may have benefited from the construction of patient‐specific subpathway activities. We compared the prognostic value of the four‐subpathway signature with that of the MammaPrint gene signature, which is one of the most common clinically used gene‐based models for breast cancer prognosis prediction. The predictive performance of the four‐subpathway signature was superior to that of the MammaPrint signature across the breast cancer data sets used in our study based on the log‐rank test and time‐dependent ROC analysis (Figures S6 and S7). The subpathway signature, which incorporates higher‐order information of biological pathways, may be the reason for the production of more robust results than the gene‐based signature.

We also found that there were only three overlapping genes between the MammaPrint signature and our subpathway signature. We then test if there are more overlaps at the pathway level instead of gene level. The 70 genes in the MammaPrint signature were then mapped to the KEGG pathway database. Twenty‐five pathways were annotated with at least one gene, of which five are statistically significant (hypergeometric test, *P *<* *.05). Interestingly, we found that the four subpathways in our signature are all ranked top ten in the 25 MammaPrint pathway list. Moreover, there are two overlapped pathways (Hippo signalling pathway and PI3K‐Akt signalling pathway) between five MammaPrint pathways and four subpathways in our signature. Through hypergeometric test on the two pathway set, the statistically significant *P*‐value reached .002. These results indicate that the MammaPrint signature and our subpathway signature are quite consistent in pathway level and may perform some similar biological function.

Although the four‐subpathway signature was proposed to predict the overall survival of various patients with breast cancer, we wanted to know whether the signature could classify patients with the same subtype into high‐ and low‐risk groups with significantly different survival prospects. Learning about the different subtypes of breast cancer can be very helpful for understanding treatment options and prognosis. Recently, ER+ breast cancer is one of the most common types of breast cancer diagnoses. Patients with ER+ breast cancer have a generally good prognosis for 5‐year survival,^30^ but this outcome is not always the case. In our stratified analysis, the four‐subpathway signature effectively classified patients with ER+ into high‐ and low‐risk groups across four data sets (Figure 3A‐D). This result indicates that the subpathway signature may help doctors to identify high‐risk patients in the ER+ cohort, which may further lead to tailored treatment. For patients with the ER− subtype, which generally have a poor prognosis in clinical practice,^31^ a high proportion of these patients was classified into high‐risk groups by the prognostic value of the four‐subpathway signature, which resulted in a non‐significant *P* in the log‐rank test. Thus, a more specific signature for classifying patients of breast cancer with the ER− subtype remains to be developed.

We further compared the survival prediction power between clinical characteristics and the four‐subpathway signature. The results showed that the four‐subpathway signature displayed better predictions of survival than age or tumour size in regard to overall survival across five sets based on time‐dependent ROC analysis (Figure 4). Thus, we expect that the four‐subpathway signature can assist doctors in clinical practice.

Currently, one limitation of the method is that it classifies samples into high‐risk or low‐risk group according to the median risk score in the training set, which is not interpretable in clinical settings. This is a common limitation for the current bioinformatics method for identifying prognosis signature based on high‐throughput experimental data. Another limitation is that normally prognosis is defined as 5‐year survival or 10‐year survival; however, the data for all patients with breast cancer with follow‐up information at least 5 year or 10 year are generally unavailable. Instead, we defined poor or good prognosis according to the status of patient death or not in the study. We believe that the prognosis classification performance of the methods will be considerably improved once we have a more complete survival data. To make our method clinically usable, some essential biological experiments need to be performed to test the association between the four‐subpathway signature and pathogenic mechanism of breast cancer.

## CONFLICT OF INTEREST

The authors declare no competing financial interests.
