# Supplementary material for: Inference of patient‐specific subpathway activities reveals a functional signature associated with the prognosis of patients with breast cancer
Source: J Cell Mol Med. 2018 Jul 4;22(9):4304–16. doi: 10.1111/jcmm.13720 (PMC6111825; doi:10.1111/jcmm.13720)
Supplement: Supplementary file 1 [file JCMM-22-4304-s001.pdf]

Supporting Information

Inference of patient-specific subpathway activities reveals a functional signature associated with the prognosis of patients with breast cancer

Inventory of Supporting Information

1. Appendix S1
2. Table S1-S6
3. Figure S1-S11

Appendix S1

The four-subpathway signature has prognostic value within HER2 subtypes of breast cancer

We did stratified analyses of patients with HER2＋ and HER2－ patients from the TCGA and GSE1992 datasets. Compared with patients with low-risk scores, patients with HER2－ disease and high-risk scores had shorter overall survival (p=0.00036 in TCGA and p=0.0007 in GSE1992) (Supplementary Figure S4A, C). HER2＋ is an aggressive subtype of breast cancer, and patients with HER2＋ tend to have a poor prognosis [1]. In the TCGA and GSE1992 data sets, we also found that patients with HER2＋ have shorter survival times than patients with HER2－ (Supplementary Figure S4B, D). The subpathway signature classified a high proportion of patients with HER2＋ into high-risk groups; thus, the *p* of the log-rank test was not significant in the cohort of HER2＋patients (Supplementary Figure S4B, D).

Functional analysis of the subpathways

We next sought to explore the biological function of the subpathways of the prognostic signature in breast cancer tumorigenesis and development. For path:04390_17 in the hippo signaling pathway, genes in the subpathways were mapped to the original pathway, and a local region was identified (Supplementary Figure S9). This local region corresponds to key pathway downstream transcription co-activators: YAP/TAZ, which has been reported to be associated with regulating cell growth, differentiation, and apoptosis [2,3]. Specifically, elevated yes associated protein 1 (YAP1) activity has been correlated to a poor prognosis in several cancers [4,5], TEA domain transcription factor 1 (TEAD1) has been shown to be the major

YAP1 partner in breast cancer cell lines [6], and fibroblast growth factor 1 (FGF1) plays an important role in the regulation of cell survival, cell division, angiogenesis, and cell differentiation [7]. The other three subpathways have also been reported to be associated with the development of breast cancer (see Supplementary Text).

References

1. **Masood S, Bui MM.** Prognostic and predictive value of HER2/neu oncogene in breast cancer. *Microscopy research and technique*. 2002; 59: 102-8.
2. **Zhang K, Qi HX, Hu ZM, Chang YN, Shi ZM, Han XH, Han YW, Zhang RX, Zhang Z, Chen T, Hong W.** YAP and TAZ Take Center Stage in Cancer. *Biochemistry*. 2015; 54: 6555-66.
3. **Moroishi T, Park HW, Qin B, Chen Q, Meng Z, Plouffe SW, Taniguchi K, Yu FX, Karin M, Pan D, Guan KL.** A YAP/TAZ-induced feedback mechanism regulates Hippo pathway homeostasis. *Genes & development*. 2015; 29: 1271-84.
4. **Xu MZ, Yao TJ, Lee NP, Ng IO, Chan YT, Zender L, Lowe SW, Poon RT, Luk JM.** Yes-associated protein is an independent prognostic marker in hepatocellular carcinoma. *Cancer*. 2009; 115: 4576-85.
5. **Hall CA, Wang R, Miao J, Oliva E, Shen X, Wheeler T, Hilsenbeck SG, Orsulic S, Goode S.** Hippo pathway effector Yap is an ovarian cancer oncogene. *Cancer research*. 2010; 70: 8517-25.
6. **Zhao B, Ye X, Yu J, Li L, Li W, Li S, Yu J, Lin JD, Wang CY, Chinnaiyan AM, Lai ZC, Guan KL.** TEAD mediates YAP-dependent gene induction and growth control. *Genes & development*. 2008; 22: 1962-71.
7. **Mori S, Tran V, Nishikawa K, Kaneda T, Hamada Y, Kawaguchi N, Fujita M, Saegusa J, Takada YK, Matsuura N, Zhao M, Takada Y.** A dominant-negative FGF1 mutant (the R50E mutant) suppresses tumorigenesis and angiogenesis. *PloS one*. 2013; 8: e57927.

Table S1. Summary of patient and tumor characteristics.

Characteristics	Training set in Vijver data	Test set in Vijver data	GSE1992	GSE7390	TCGA	GSE 1456	GSE3143
Microarray platform	Agilent	Agilent	Agilent G4110A	Affymetrix HG-U133A	Agilent G4502A	Affymetrix HG-U133A	Affymetrix HG-U95A
No. of patients (%)	147	148	161	198	531	159	158
Dead	41 (27.9%)	38 (25.7%)	26 (16.1%)	56 (28.3%）	65 (12.2%)	40 (25.2%)	75 (47.5%)
Censored	106 (72.1%)	110 (74.3%)	93 (57.8%)	142 (71.7%)	392 (73.8%)	119 (74.8%)	83 (52.5%)
NA			42(26.1%)		74(14%)		
Age, median (year)	45	44	55	46	58	—	—
Tumor size (%)							
<=2cm	74 (50.3%)	81 (54.7%)	30 (18.6%)	102 (51.5%)	133 (25%)	—	—
>2cm	73 (49.7%)	67 (45.3%)	90 (55.9%)	96 (48.5%)	392 (73.8%)	—	—
NA	—	—	41(25.5%)	—	6(1.2%)	—	—
LN status (%)							
Negative	68 (46.3%)	83 (56.1%)	50 (31.1%)	198 (100%)	268 (50.5%)	—	—
Positive	79 (53.7%)	65 (43.9%)	71 (44.1%)	—	260 (49%)	—	—
NA	—	—	40 (24.8%)	—	3 (0.5%)	—	—
Grade (%)							
1	37 (25.2%)	38 (25.7%)	10 (6.2%)	30 (15.2%)	—	—	—
2	44 (30%)	57 (38.5%)	44 (27.3%)	83 (41.9%)	—	—	—
3	66 (44.8%)	53 (35.8%)	63 (39.2%)	83 (41.9%)	—	—	—
NA	—	—	44 (27.3%)	2 (1%)		—	—
ER status (%)							
Positive	110 (74.8%)	116 (78.4%)	70 (43.4%)	134 (67.7%)	404 (76.1%)	—	—
Negative	37 (25.2%)	32 (21.6%)	50 (31.1%)	64 (32.3%)	118 (22.2%)	—	—
NA	—	—	41 (25.5%)	—	9 (1.7%)		

**HER2 status (%)**							
**Positive**	—	—	32 (19.9%)	—	76 (14.3%)	—	—
**Negative**	—	—	109 (67.7%)	—	433 (81.5%)	—	—
**NA**	—	—	20 (12.4%)	—	22 (4.2%)	—	—
**Stage (%)**							
**I**	—	—	—	—	91 (17.1%)	—	—
**II**	—	—	—	—	296 (55.7%)	—	—
**III**	—	—	—	—	109 (20.5%)	—	—
**IV**	—	—	—	—	14 (2.6%)	—	—
**Median survival (months)**	86.2	88.7	84.6	47.4	23	151.5	27.9

Abbreviation: LN, lymph node; ER, estrogen receptor; HER2, human epidermal growth factor receptor 2.

"—"represents that data is not provided.

Table S2. Nine subpathways mostly related to the prognostic classification

SubpathwayID	Pathway	Subpathway Size	Genes in the Subpathway	Univariate cox regression p-values	The rank of the subpathway in the 922 subpathway list
path04024_59	cAMP signaling pathway	8	CALM1, ADCY3, RAPGEF3, GNAI1, MAPK10, GNAS, GABBR2, HTR6	4.19e-09	51
path04390_17	Hippo signaling pathway	8	YWHAZ, YWHAG, YAP1, SOX2, SERPINE1, TEAD3, BIRC5, FGF1	4.83e-10	5
path04630_47	Jak-STAT signaling pathway	8	MCL1, STAT5A, BCL2, JAK2, IL20RA, IL3RA, IL2RG, IL23A	6.65e-08	233
path04730_1	Long-term depression	10	LYN, PRKCA, PLA2G4B, GNAS, GNAZ, GNA12, CRHR1, GRM1, GNAQ, GNAI1	2.59e-09	37
path05200_96	Pathways in cancer	8	RALBP1, RAC3, MAPK10, RALA, PLD1, RALGDS, HRAS, PRKCG	2.06e-09	28
path04151_102	PI3K-Akt signaling pathway	9	GH2, JAK2, IL2RG, PIK3CD, IRS1, IL7R, IGF1R, FGF10, FGF18	1.20e-07	306
path00230_30	Purine metabolism	8	PDE1A, GMPS, ITPA, POLR3A, PDE2A, ENTPD2, RRM2, ADCY7	1.40e-08	112
path04015_41	Rap1 signaling pathway	9	PIK3CD, RAP1B, RAPGEF6, SKAP1, SIPA1L1, PLCE1, MAGI1, CTNNB1, PIK3CG	1.44e-07	334
path04015_117	Rap1 signaling pathway	8	FGF7, EGFR, VEGFA, FGF11, PDGFRB, FGF3, FGF20, FGFR4	1.07e-06	555

Table S3. Univariate Cox proportional hazard regression analysis for the four-subpathway signature in the seven data sets.

Data sets	HR	95% CI	p-value
Training set	1.90	1.57-2.31	5.10E-11
Test set	1.25	1.13-1.38	9.12E-06
GSE1992	1.37	1.15-1.62	0.00039
GSE7390	1.22	1.09-1.37	0.00062
TCGA	2.02	1.22-3.35	0.0065
GSE1456	1.20	1.04-1.37	0.010
GSE3143	1.29	1.07-1.54	0.0072

Table S4. Clinical and pathological characteristics of patients with breast cancer with high- or low-risk subpathway signature in the three independent sets.

Characteristics	GSE1992 (N=161)			GSE7390 (N=198)			TCGA (N=531)		
	High-risk group (n=78)	Low-risk group (n=83)	*p*-value	High-risk group (n=93)	Low-risk group (n=105)	*p*-value	High-risk group (n=254)	Low-risk group (n=277)	*p*-value
Age, median (sd)	50(13.69)	58(15.72)	0.005*	46(7.28)	47(7.15)	0.24*	55(13.47)	61(12.75)	0.002*
Size (%)			0.056			0.33			<0.001
<=2cm	8(10.26)	22(26.51)		44(47.31)	58(55.24)		41(16.14)	92(33.21)	
>2cm	44(56.41)	46(55.42)		49(52.69)	47(44.76)		21（82.68）	18（65.70）	
LN status (%)			0.18			N/A			0.03
Positive	34(43.59)	37(44.58)		0	0		119(46.85)	109(39.35)	
Negative	17(21.79)	33(39.76)		93	105		97(38.19)	136(49.10)	
Grade, No. (%)			<0.001			<0.001			—
1	0（0）	10(12.05)		8(8.60)	22(20.95)		—	—	
2	14(17.95)	30(36.14)		26(27.96)	57(54.29)		—	—	
3	38(48.72)	25(30.12)		58(62.37)	25(23.81)		—	—	
ER status (%)			<0.001			<0.001			<0.001
Positive	15(19.23)	55(66.27)		42(45.16)	92(87.62)		147(57.87)	257(92.78)	
Negative	37(47.44)	13(15.66)		51(54.84)	13(12.38)		103(40.55)	15(5.42)	
Her2 status (%)			0.01			—			<0.001
Positive	22(28.21)	10(12.05)		—	—		61(24.02)	15(5.42)	
Negative	45(57.69)	64(77.11)		—	—		179(70.47)	254(91.70)	
Median survival (months)	22	24	<0.001^{+}	137.8	156.4	<0.001^{+}	27.63	28.53	<0.001^{+}

Abbreviation: LN, lymph node; ER, estrogen receptor; Her2, human epidermal growth factor receptor 2.

P-values are calculated by chi-square test, unless otherwise stated. *Student’s t-test; $^{+}$Log-rank test.

"—" represents that data is not provided. N/A represents that p-values are not calculated because all patients are LN negative.

Table S5. Multivariate Cox proportional hazards regression analysis of the subpathway signature and clinical characteristics

Variable	Training set		Test set		GSE1992		GSE7390		TCGA	
	**HR (95% CI)**	***p*-value**	**HR (95% CI)**	***p*-value**	**HR (95% CI)**	***p*-value**	**HR (95% CI)**	***p*-value**	**HR (95% CI)**	***p*-value**
Age	2.63 (1.05-6.60)	0.04	0.64 (0.19-2.14)	0.47	2.07 (0.85-4.97)	0.11	1.42 (0.80-2.52)	0.23	1.70 (1.06-2.75)	0.03
Tumor size	1.01 (0.48-2.12)	0.97	1.81 (0.89-3.69)	0.10	2.13 (0.93-4.85)	0.07	1.20 (0.69-2.06)	0.51	1.17 (0.60-2.27)	0.64
ER status	2.95 (1.26-6.91)	0.01	0.56 (0.26-1.23)	0.15	0.73 (0.24-2.25)	0.58	0.68 (0.34-1.37)	0.28	1.63 (0.77-3.48)	0.20
Grade	1.18 (0.56-2.46)	0.67	2.62 (1.27-5.40)	0.01	3.39 (1.11-10.34)	0.03	0.73 (0.38-1.42)	0.35	—	—
Her2 status	—	—	—	—	1.46 (0.58-3.65)	0.42	—	—	0.66 (0.30-1.49)	0.32
LN status	0.78 (0.41-1.48)	0.45	0.79 (0.39-1.57)	0.49	4.19 (1.32-12.22)	0.01	N/A	N/A	1.66 (0.94-2.92)	0.07
4-subpathway	1.90 (1.57-2.31)	5.10E-11	1.14 (1.0-1.29)	0.05	1.28 (1.03-1.57)	0.02	1.21 (1.05-1.40)	0.009	1.79 (1.05-3.03)	0.03

Abbreviation: ER, estrogen receptor, Her2, human epidermal growth factor receptor 2; LN, lymph node.

"—"represents that data is not provided; N/A represents that HR and p values are not calculated because all patients are LN negative.

Table S6. The p-value of log-rank survival analysis of the four-subpathway and the less-than-four-subpathway signatures in the training set, test set, and five independent sets.

Signatures	Training set	Test set	GSE1992	GSE7390	TCGA	GSE1456	GSE3143
**4-subpathway signature**	1.8E-13	4.16E-06	0.00022	0.00025	0.00067	0.030	0.029
**3-subpathway signature**							
path:04390_17+ path:04730_1+ path:04151_102	3.6E-13	4.8E-05	0.00084	0.01	0.0052	0.036	0.059
path:04390_17+ path:04151_102+ path:00230_30	5.3E-12	2.0E-06	0.00051	0.00050	0.00082	0.01	0.060
path:04390_17+ path:04730_1+ path:00230_30	6.6E-11	0.00016	0.042	0.0021	0.30	0.16	0.57
path:04730_1+ path:04151_102+ path:00230_30	2.7E-13	1.1E-05	0.00014	0.0027	0.042	0.30	0.054
**2-subpathway signature**							
path:04390_17+ path:04730_1	7.9E-08	0.0032	0.23	0.048	0.86	0.13	0.83
path:04390_17+ path:04151_102	1.0E-09	1.5E-05	0.0077	0.070	0.0016	0.075	0.13
path:04390_17+ path:00230_30	4.8E-12	1.3E-05	0.015	0.0024	0.11	0.0030	0.046
path:04730_1+ path:04151_102	1.3E-10	0.0098	4.5E-05	0.00053	0.006	0.607	0.21
path:04730_1+ path:00230_30	2.5E-07	0.00072	0.042	0.0094	0.97	0.57	0.20
path:04151_102+ path:00230_30	2.0E-09	1.2E-06	0.0016	0.0043	0.015	0.034	0.026
**1-subpathway signature**							
path:04390_17	4.7E-07	0.00042	0.044	0.027	0.34	0.018	0.092
path:04730_1	0.00093	0.43	0.23	0.071	0.60	0.47	0.47
path:04151_102	1.9E-07	0.037	0.00052	0.11	0.00036	0.028	0.44
path:00230_30	1.2E-10	4.2E-07	0.051	0.073	0.20	0.053	0.28

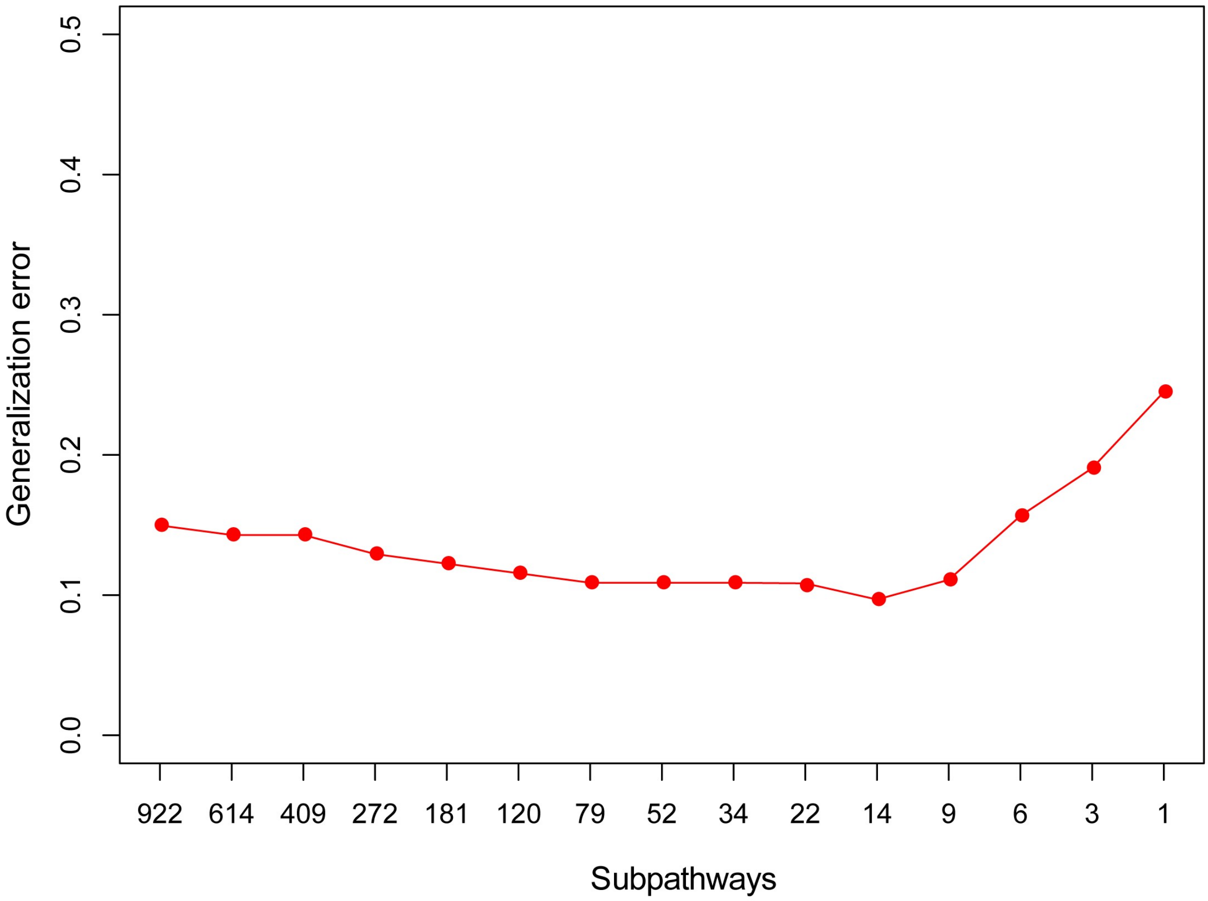

Figure S1. The generalization error of random forest classification algorithm estimated by the retained subpathways after each deletion.

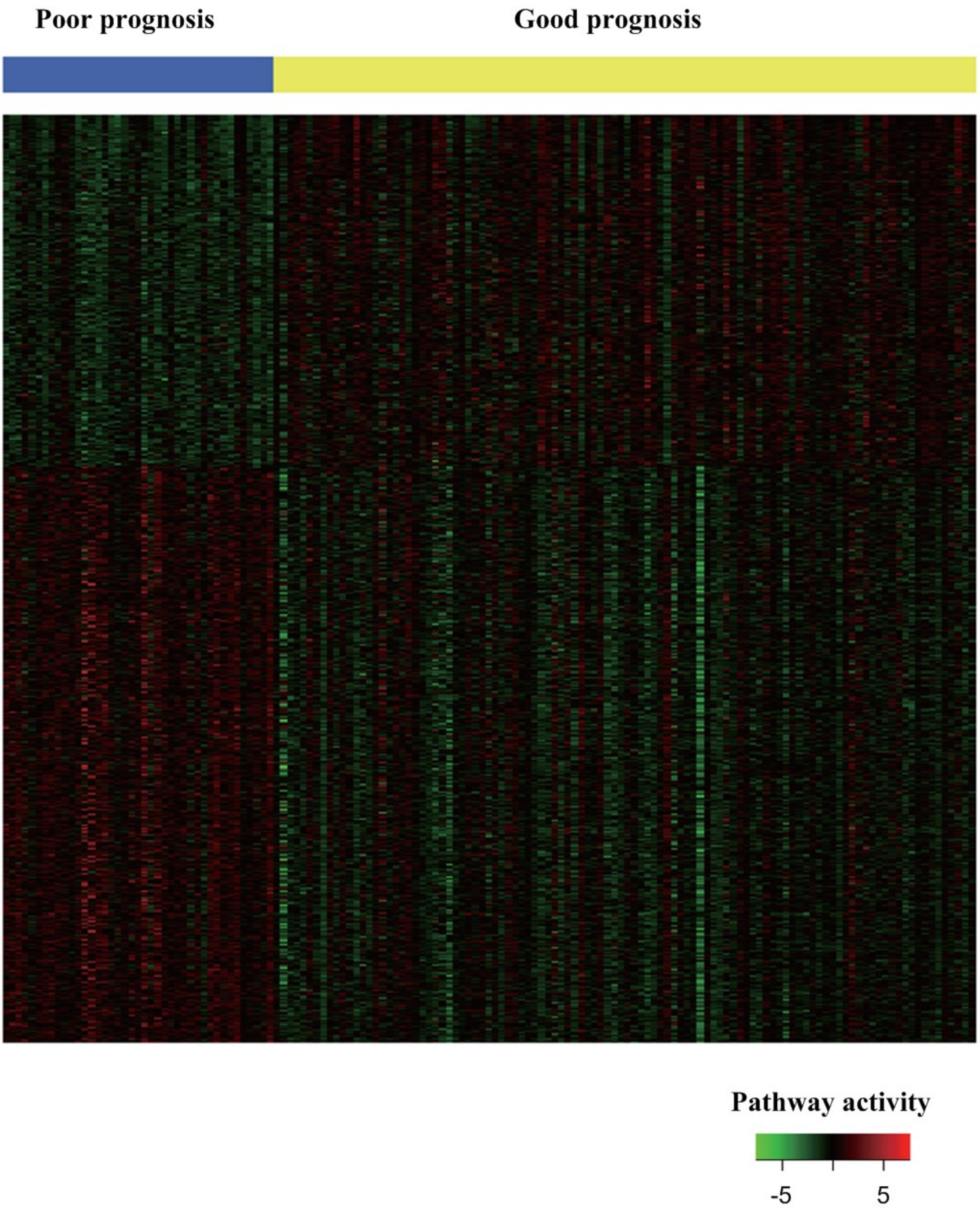

Figure S2. Heat map of the activities of 922 subpathways between patients with good prognosis and poor prognosis in the training set.

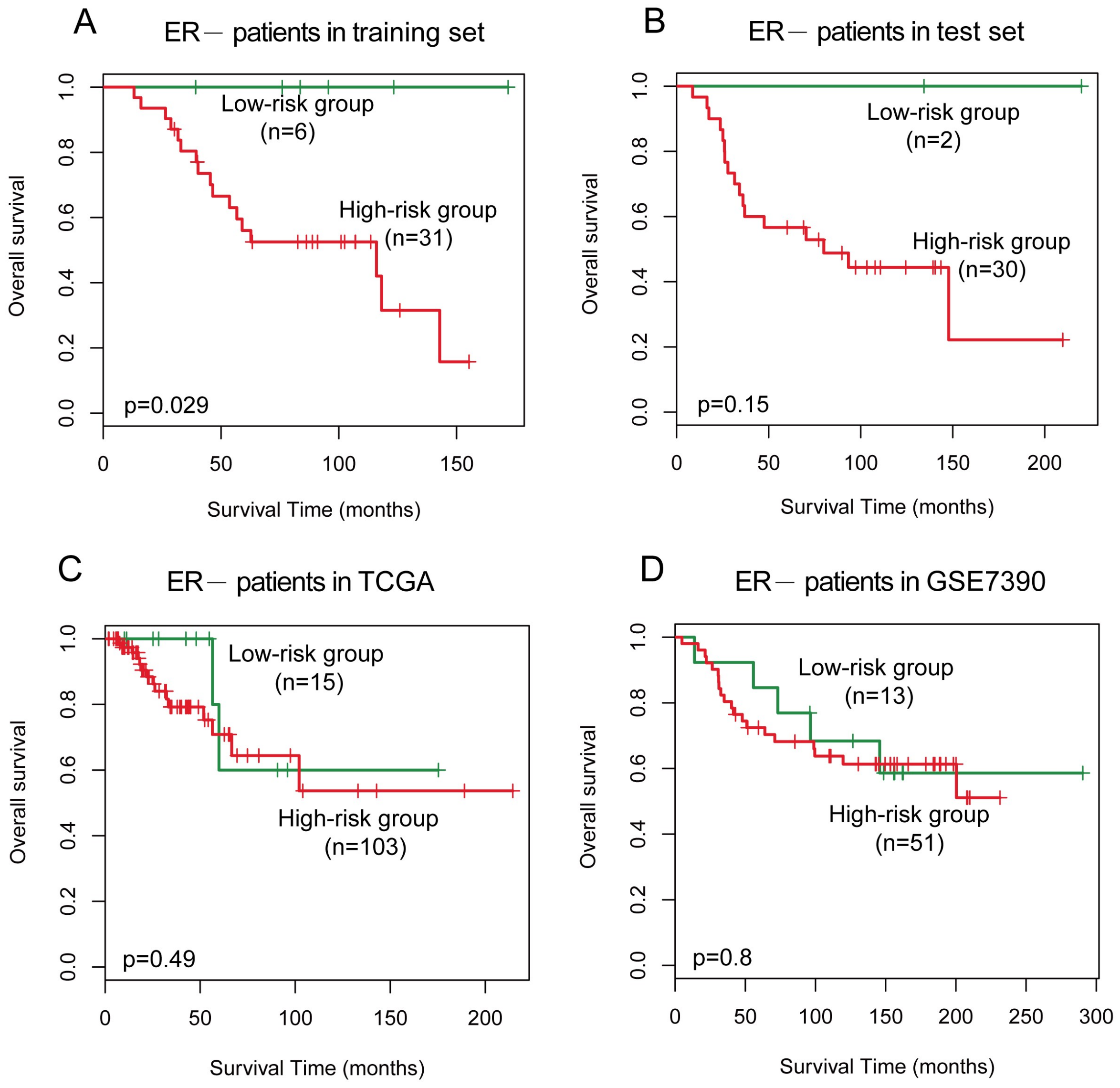

Figure S3. Survival prediction in ER－ patients. Kaplan-Meier survival curves of ER－ patients with breast cancer classified into high- and low-risk groups based on the four-subpathway signature. (A) Training set (n=37). (B) Test set (n=32). (C) TCGA (n=118). (D) GSE7390 (n=64).

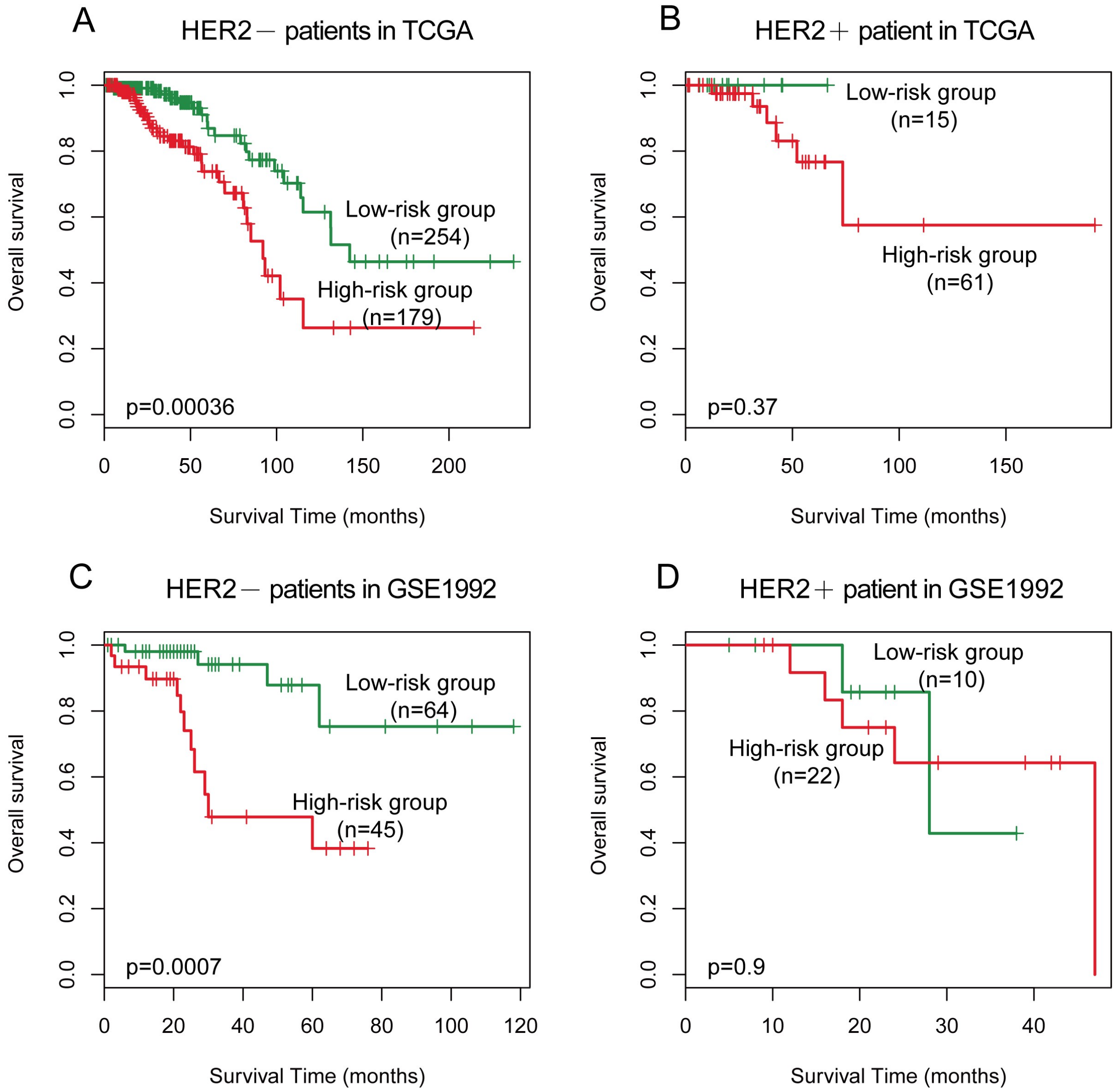

Figure S4. Survival prediction in HER2＋ and HER2－ patients. Kaplan-Meier survival curves of HER2＋ and HER2－ patients with breast cancer classified into high- and low-risk groups based on the four-subpathway signature. (A) HER2－ patients, TCGA (n=433). (B) HER2＋ patients, TCGA (n=76). (C) HER2－ patients, GSE1992 (n=109). (D) HER2＋ patients, GSE1992 (n=32). Vertical hash marks indicate censored data.

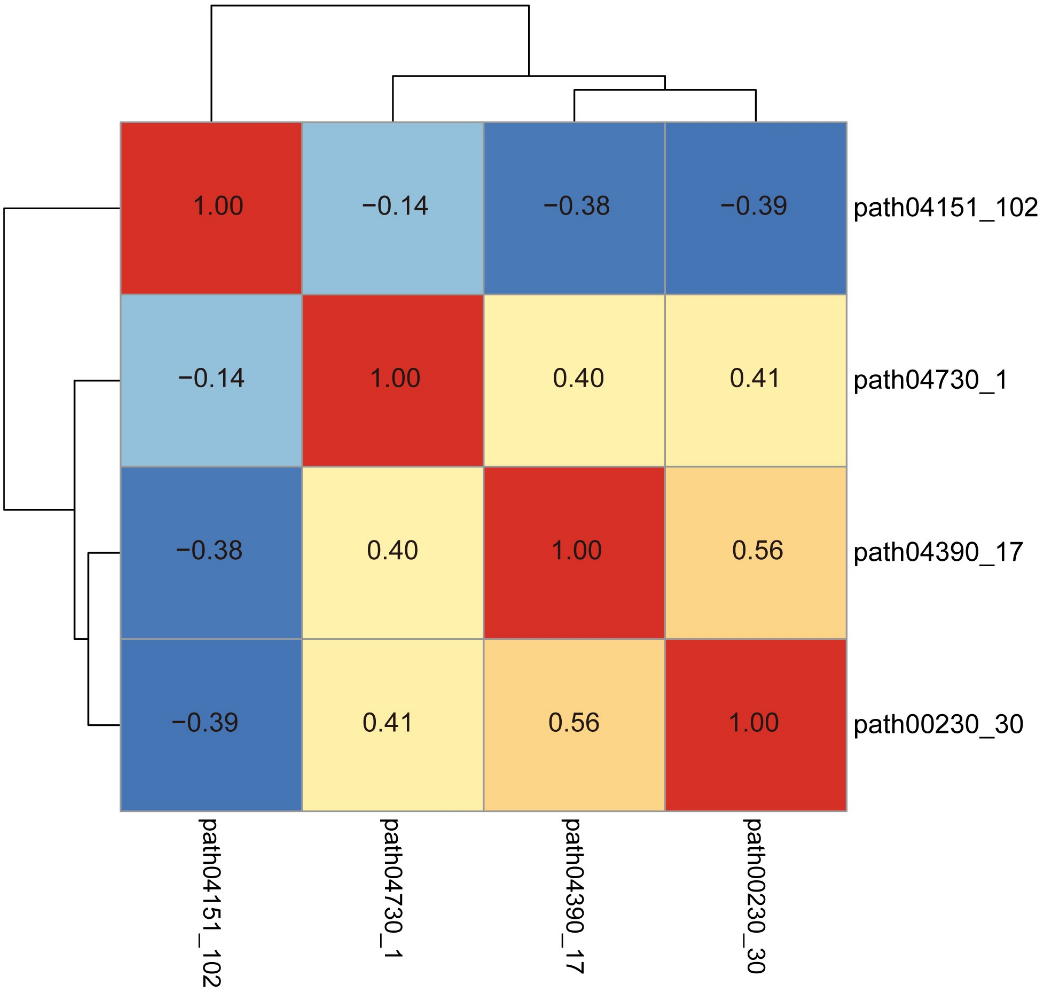

Figure S5. Pearson correlation coefficient matrix between the four subpathway activities

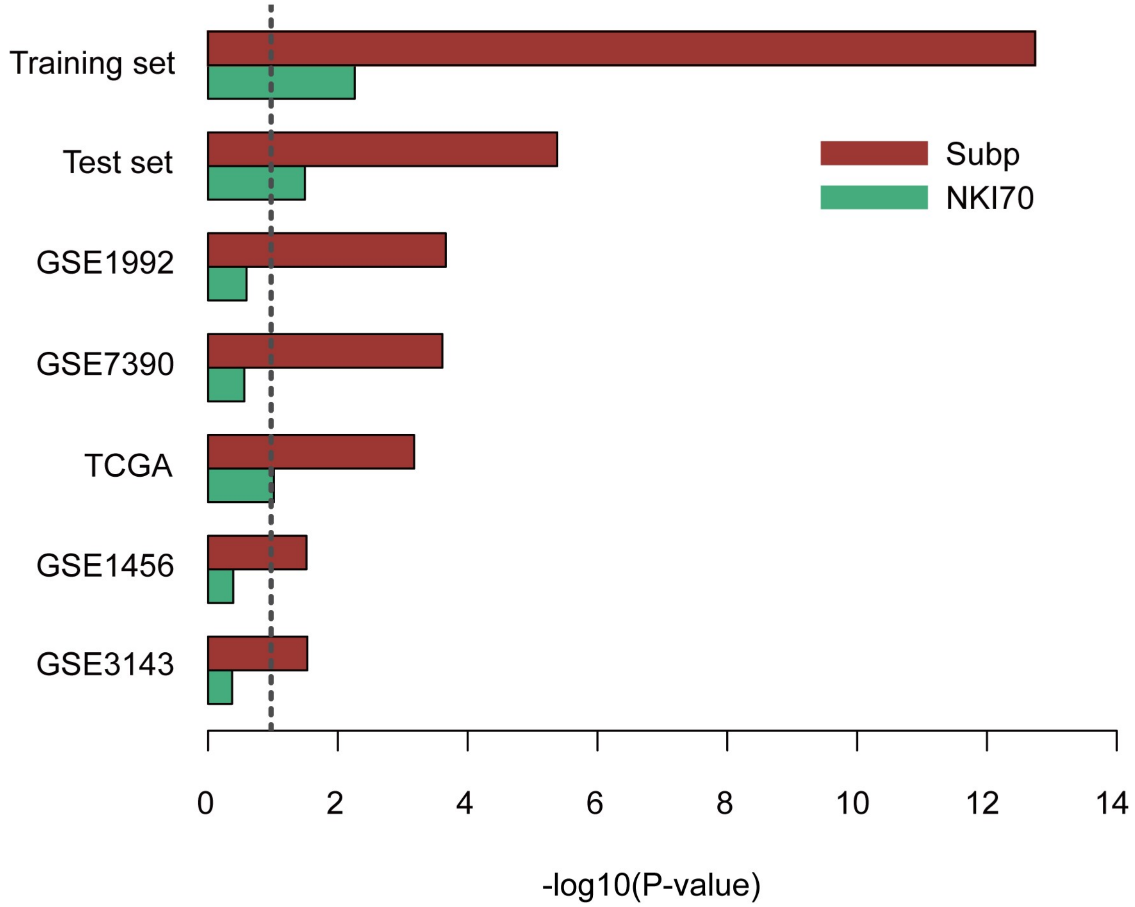

Figure S6. Comparisons of the *p*-values of log-rank test in Kaplan-Meier method for the prediction of survival by the four-subpathway signature (Subp) and NKI70 signature (NKI70). Dash line corresponds to -log10(*p*)=1.3 (*p*=0.05).

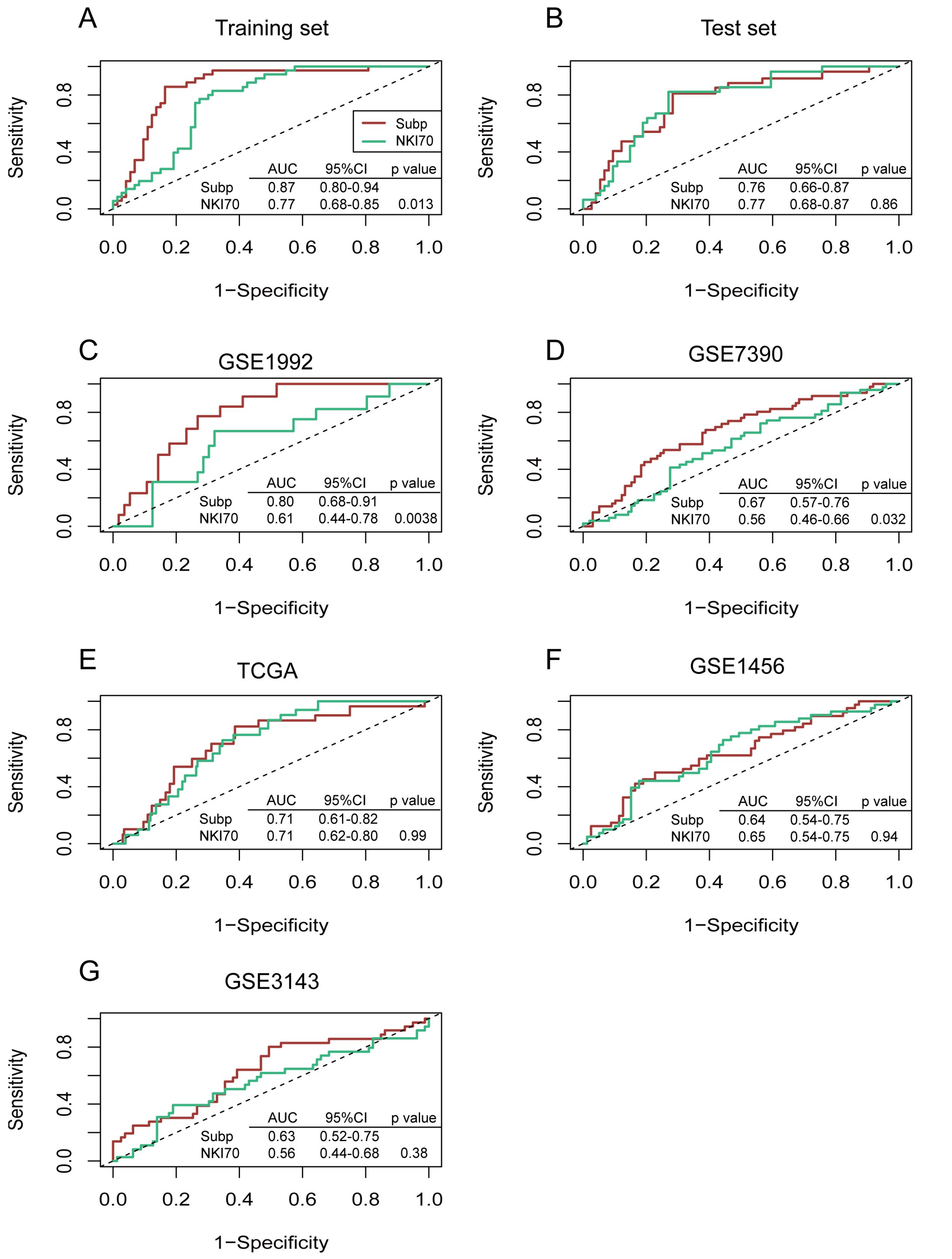

Figure S7. Comparisons of the sensitivity and specificity for the prediction of overall survival by the four-subpathway signature (Subp) and NKI70 signature (NKI70). Receiver operating characteristics (ROC)

curves in the (A) training set, (B) test set, (C) GSE1992, (D) GSE7390, (E) TCGA, (F) GSE1456, and (G) GSE3143. P-values show the area under the ROC (AUC) of the four-subpathway signature versus AUCs of the NKI70 signature.

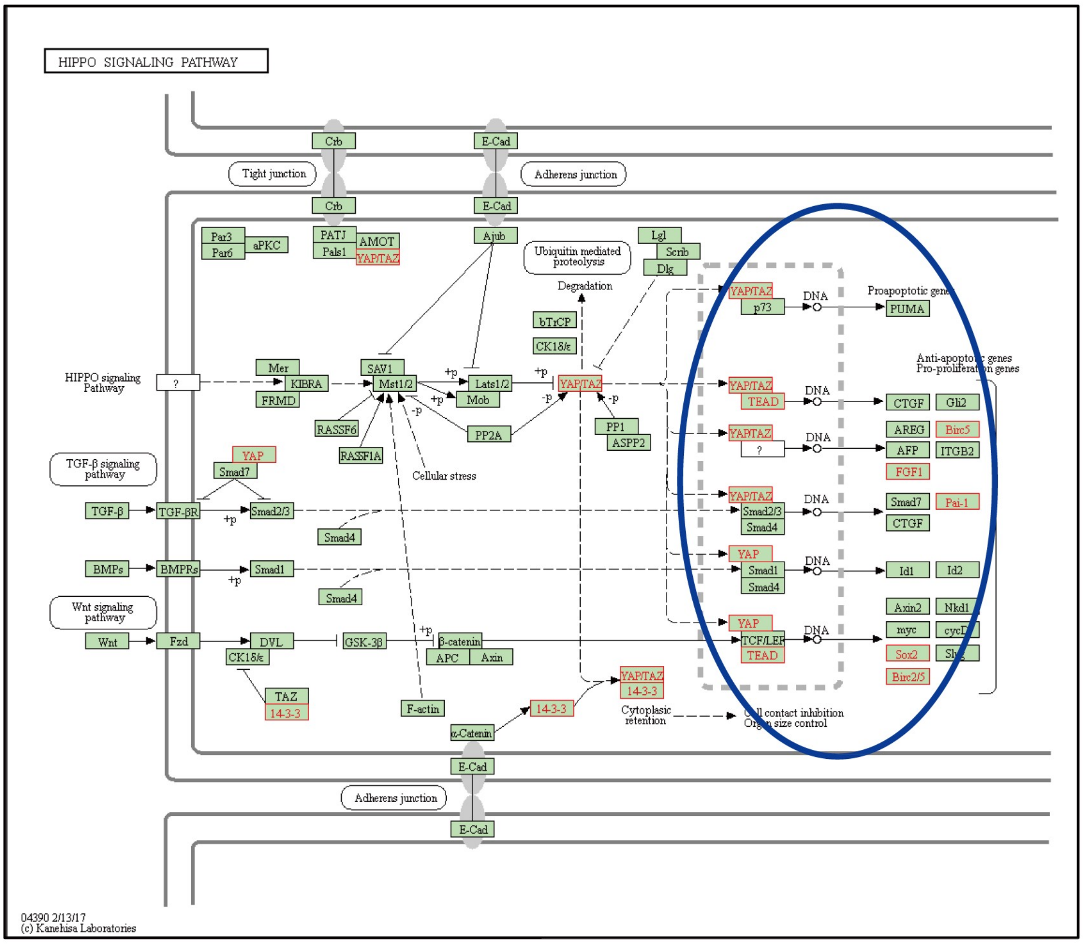

Figure S8. Annotation of genes in the path:04390_17 subpathway to the original hippo signaling pathway in KEGG. Genes are marked with red, and the blue circle corresponds to the local region of the subpathway.

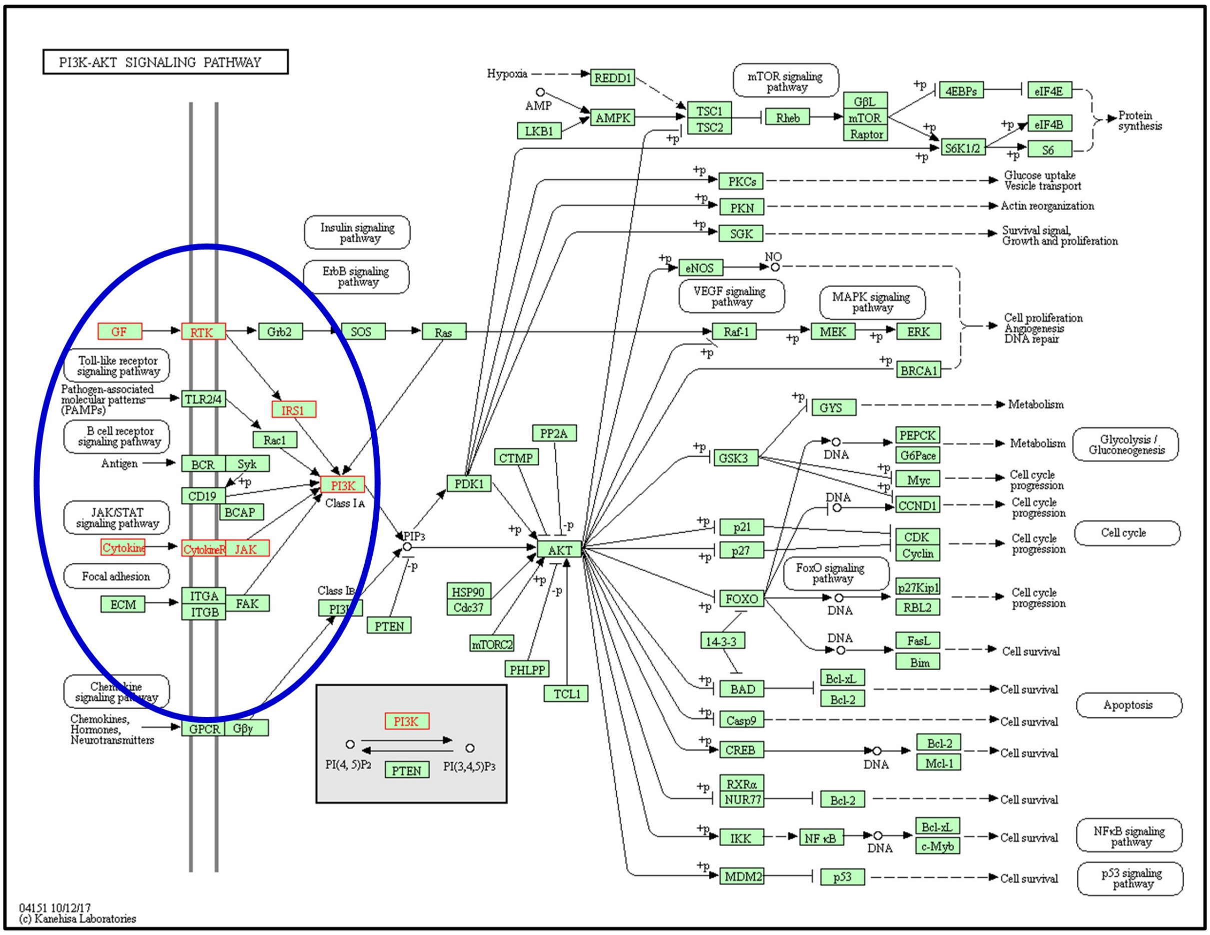

Figure S9. Annotation of genes in the path:04151_102 subpathway to the original PI3K-Akt signaling pathway in KEGG. Genes are marked with red, and the blue circle corresponds to the local region of the subpathway.

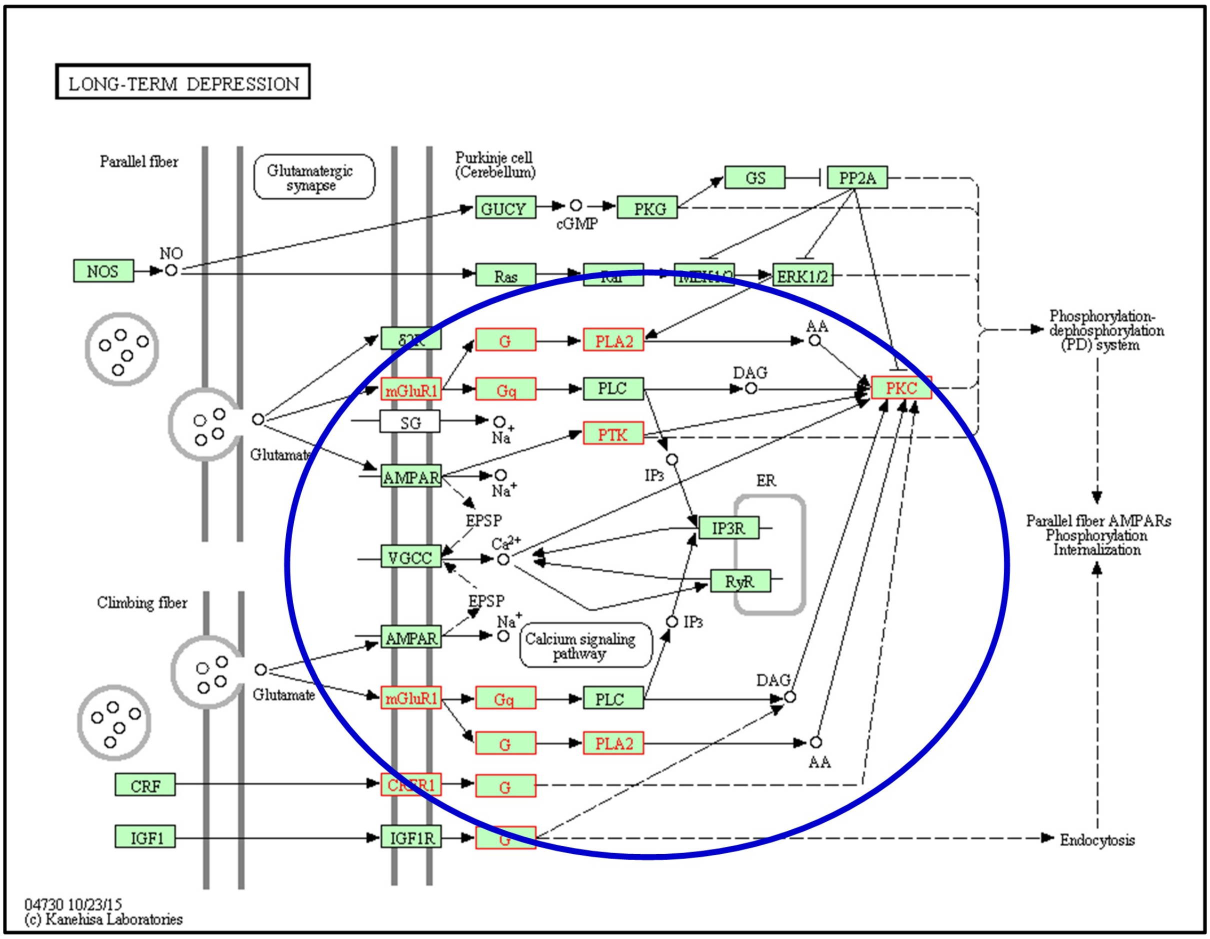

Figure S10. Annotation of genes in the path:04730_1 subpathway to the original Long-term depression pathway in KEGG. Genes are marked with red, and the blue circle corresponds to the local region of the subpathway.

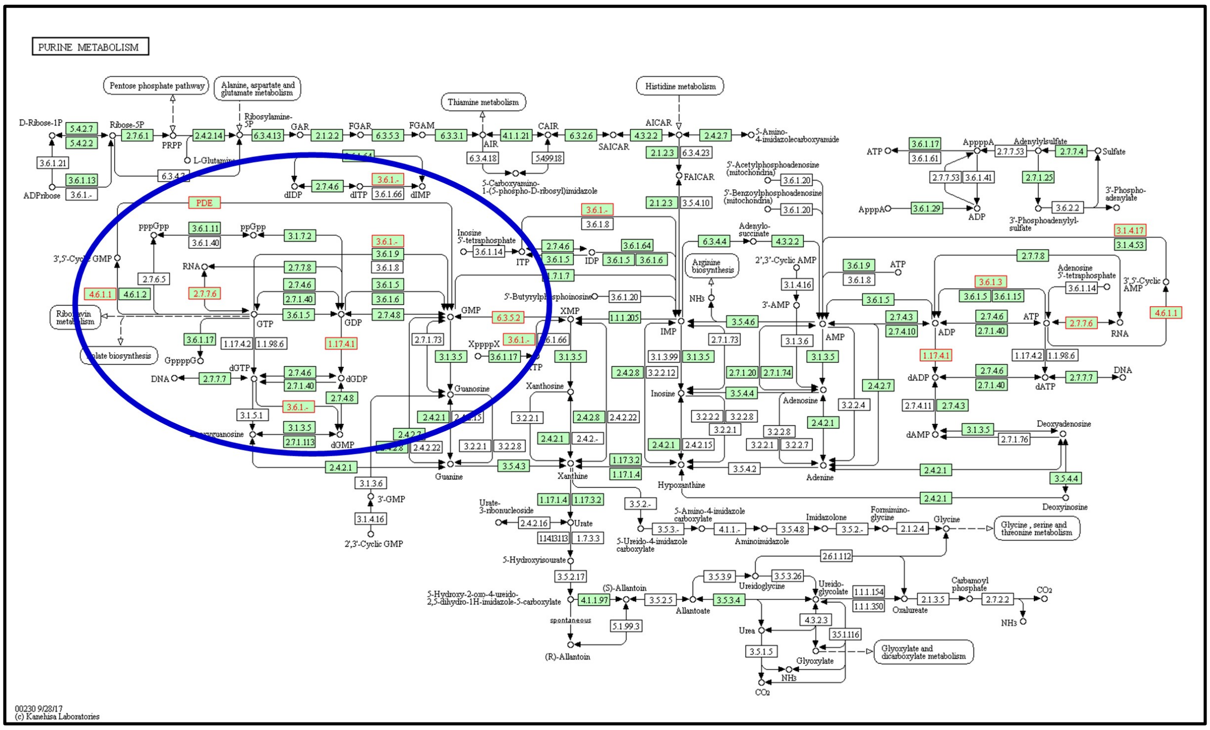

Figure S11. Annotation of genes in the path:00230_30 subpathway to the original Purine metabolism pathway in KEGG. Genes are marked with red, and the blue circle corresponds to the local region of the subpathway.
